# Microbiome-driven precision psychiatry: integrating multi-omics, artificial intelligence, and personalized interventions along the gut-brain axis

**DOI:** 10.3389/fmed.2026.1957715

**Published:** 2026-10-15

**Authors:** Yi Zhang, Changyong Pei, Daohua Zhang, Jun Liu

**Affiliations:** 1 General Practice Department, The Second People's Hospital of Changde City, Changde, China; 2 Department of General Practice, The First Affiliated Hospital of Changde Vocational and Technical College, Changde, China; 3 Medical Affairs Department, The Second People's Hospital of Changde City, Changde, China; 4 Department of General Medicine, Xiangya Hospital, Central South University, Changsha, China

**Keywords:** lipidomics, metabolomics, metagenomics, microbiome-driven precision medicine, microbiota-gut-brain axis, multi-omics integration

## Abstract

**Introduction:**

This scoping review maps evidence for microbiome-driven precision psychiatry in depression and anxiety.

**Methods:**

Following JBI guidance and PRISMA-ScR, we searched PubMed/MEDLINE and PubMed Central from inception to July 2026, supplemented by Google Scholar and backward citation searching. Human primary studies were screened and charted; findings were synthesized narratively.

**Results:**

Of 58 identified records, 49 remained after deduplication, 35 full texts were assessed, and 28 studies were included. The evidence was predominantly observational. Taxonomic findings varied across populations, while studies of microbial functions, metabolites, multi-omics signatures, and treatment response suggested potential for biological stratification. Microbiome-targeted interventions and artificial intelligence models remain exploratory.

**Discussion:**

Microbiome-derived biomarkers and predictive models require standardized methods, independent validation, and prospective evidence of clinical utility before routine psychiatric use.

## Graphical Abstract

**Figure:**
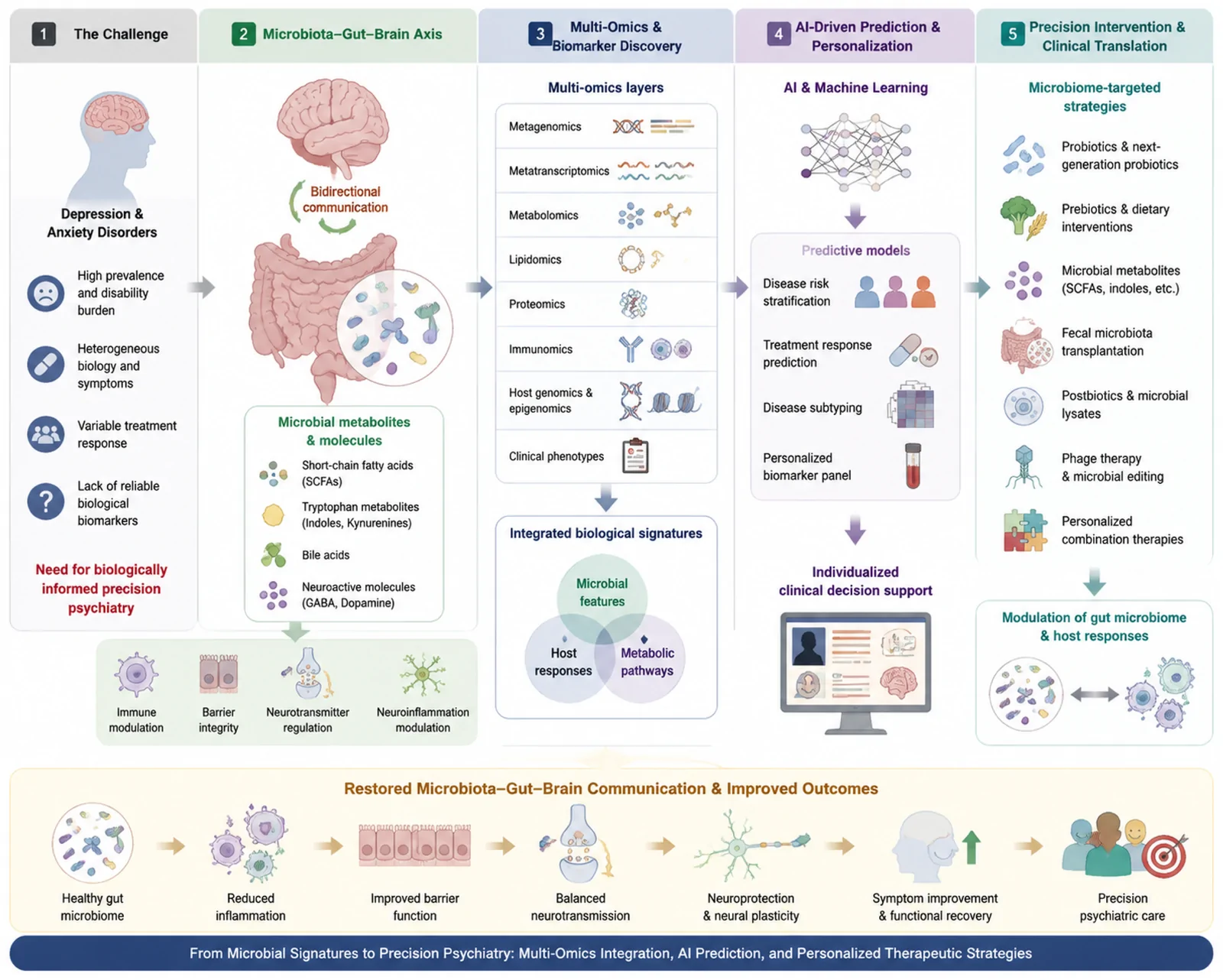

## 1 Introduction

Depression and anxiety disorders represent major contributors to global disease burden, disability, and reduced quality of life. According to the Global Burden of Disease Study 2019, depressive and anxiety disorders contribute substantially to global disability and remain major causes of years lived with disability worldwide (1). Although psychological interventions and pharmacological treatments have improved outcomes for many individuals, considerable heterogeneity persists in disease trajectories, biological mechanisms, and therapeutic responses. A substantial proportion of patients fail to achieve adequate remission despite guideline-based treatments, highlighting the limitations of psychiatric approaches that rely primarily on symptom-based classification rather than underlying biological mechanisms (2, 3). Current psychiatric diagnostic frameworks remain largely based on clinical symptoms and behavioral assessments. Precision medicine has traditionally focused on genomic variation; however, increasing evidence indicates that human health is shaped by dynamic interactions between host genetics, environmental exposures, and microbial ecosystems (3). Unlike static genomic information, the microbiome represents a modifiable biological interface capable of reflecting both environmental influences and host physiological states. Therefore, microbiome profiling provides a complementary dimension for precision medicine by capturing functional biological variation that may influence disease susceptibility, progression, and therapeutic response (2). Importantly, microbiome-mediated host interactions extend beyond the nervous system: evidence from metabolic disorders such as diabetes illustrates how dysbiosis, altered microbial metabolites, intestinal barrier dysfunction, and inflammatory signaling can influence interconnected gut-pancreas, gut-liver, gut-kidney, and other systemic axes (4).

Within this context, the gut microbiome has emerged as a promising biological component of precision psychiatry through its involvement in the microbiota-gut-brain axis, a complex bidirectional communication network connecting intestinal microorganisms with central nervous system function through metabolic, immune, endocrine, and neural pathways (5, 6). Gut microorganisms influence host physiology through the production and transformation of bioactive molecules, including short-chain fatty acids (SCFAs), tryptophan-derived metabolites, bile acid derivatives, and other microbial signaling molecules. These metabolites may regulate intestinal barrier integrity, immune homeostasis, neurotransmitter-related pathways, and neuroinflammatory processes that are relevant to psychiatric health (7, 8). Increasing evidence from human studies indicates that alterations in gut microbial ecosystems are associated with psychiatric phenotypes, particularly major depressive disorder (MDD) and anxiety disorders. Early investigations using 16S ribosomal RNA sequencing demonstrated differences in fecal microbial composition between individuals with MDD and healthy controls, providing initial evidence linking microbial ecosystem alterations with depressive phenotypes (9). Subsequent studies suggested that microbiome-associated changes may influence depression-related biology through altered microbial functions and host-microbiome metabolic interactions rather than isolated changes in individual microbial taxa (10). Large population-based investigations further demonstrated associations between microbial functional potential and mental health-related outcomes, highlighting the neuroactive capacity of the human gut microbiome and its potential role in brain-body communication (11).

Despite rapid advances, translation of microbiome research into clinical psychiatry remains challenging. One major limitation is the poor reproducibility of specific microbial taxa across different studies and populations. Differences in dietary patterns, medication exposure, geographical location, disease severity, sequencing platforms, bioinformatics pipelines, and analytical approaches contribute substantially to variability among findings (5, 12). Consequently, the existence of a universal “depression microbiome” or “anxiety microbiome” remains unsupported. Instead, current evidence increasingly suggests that functional microbial signatures, metabolic pathways, and integrated host-microbiome interaction networks may provide more robust biological information than individual microbial species alone (12).

The emergence of multi-omics technologies has created new opportunities to overcome these limitations by enabling investigation of microbiome-associated biological networks rather than isolated microbial alterations. Integration of metagenomics, metabolomics, lipidomics, transcriptomics, immune profiling, and clinical phenotyping allows researchers to examine how microbial communities influence host molecular pathways and psychiatric outcomes (13, 14). These approaches may facilitate discovery of biologically meaningful psychiatric subtypes, improve biomarker development, and provide new strategies for predicting treatment response. Furthermore, artificial intelligence (AI) and machine-learning approaches offer powerful analytical frameworks for integrating complex multidimensional datasets and identifying patterns that may support individualized psychiatric care (15). However, microbiome-driven precision psychiatry remains largely within the translational research phase. Current challenges include limited longitudinal evidence, insufficient external validation of predictive models, lack of standardized microbiome analytical pipelines, and uncertainty regarding whether microbiome-informed interventions provide clinically meaningful benefits beyond conventional psychiatric care (5, 16). Addressing these limitations will require large-scale longitudinal cohorts, harmonized multi-omics approaches, mechanistic validation studies, and prospective clinical trials designed to evaluate whether microbiome-based information can improve patient outcomes.

China represents an important emerging landscape for advancing microbiome precision psychiatry. The country possesses a large and diverse population, substantial variation in dietary and environmental exposures, rapidly expanding sequencing and computational capabilities, and increasing investment in precision medicine research. These characteristics provide unique opportunities to generate population-specific microbiome evidence and develop clinically relevant models of personalized psychiatric care. However, successful translation will require careful consideration of regional diversity, cultural factors, healthcare accessibility, and the need for validation across different populations.

A major unresolved challenge is translating microbiome-derived information from descriptive biological associations into clinically actionable precision psychiatry. Although previous reviews have extensively described microbiome alterations and individual mechanisms of microbiota-gut-brain communication, the integration of microbiome biology with multi-omics profiling, artificial intelligence, biomarker development, and individualized interventions within a unified precision psychiatry framework remains insufficiently synthesized. Addressing this gap requires moving beyond isolated taxonomic associations toward functional host-microbiome profiles that can be evaluated for biological stratification, treatment-response prediction, and ultimately clinical translation. Accordingly, the primary objective of this JBI-guided scoping review is to map the current evidence supporting microbiome-driven precision psychiatry in depression and anxiety disorders. Specifically, the review evaluates: (1) microbiome alterations associated with psychiatric phenotypes; (2) microbial functional pathways, metabolites, and host-microbiome interactions; (3) the contribution of multi-omics approaches to biological stratification; (4) microbiome-derived biomarkers and artificial intelligence-based predictive approaches; (5) microbiome-targeted individualized interventions; and (6) methodological and clinical barriers to translation, including considerations relevant to emerging precision-medicine settings such as China. These objectives were operationalized through the specific research questions presented in Section 2.2.

## 2 Methods

### 2.1 Review design and methodological framework

This scoping review was conducted according to the methodological guidance developed by the Joanna Briggs Institute (JBI) for evidence synthesis and was reported in accordance with the Preferred Reporting Items for Systematic Reviews and Meta-Analyses Extension for Scoping Reviews (PRISMA-ScR) guidelines (17, 18). A completed PRISMA-ScR checklist is provided as Supplementary File S1. A scoping review design was selected because microbiome-driven precision psychiatry is a rapidly evolving interdisciplinary field characterized by substantial heterogeneity in psychiatric phenotypes, study designs, microbiome platforms, molecular measurements, computational methods, interventions, and clinical outcomes. The purpose was therefore to map the breadth and characteristics of the available evidence, identify conceptual and methodological trends, and evaluate the translational progression from microbial association studies toward multi-omics-informed patient stratification and individualized intervention. The review was structured using the JBI Population-Concept-Context framework. The population comprises individuals with depressive disorders, anxiety disorders, or clinically relevant depressive or anxiety phenotypes. The central concept included gut microbiome composition and function, host-microbiome interactions, multi-omics integration, microbiome-derived biomarkers, computational prediction, and microbiome-targeted intervention. The context included clinical, community, primary-care, and population-based settings relevant to psychiatric care and precision-medicine translation (17).

### 2.2 Review objectives and research questions

The primary objective of this review was to map current evidence regarding microbiome-driven precision psychiatry approaches for depression and anxiety disorders, with particular emphasis on microbial signatures, functional biological pathways, multi-omics integration, biomarker discovery, artificial intelligence applications, and individualized interventions. The review was guided by several research questions. First, what microbiome alterations have been reported in individuals with depression and anxiety disorders? Second, which microbial functional pathways, metabolites, and host biological mechanisms are associated with psychiatric phenotypes? Third, how are multi-omics approaches being used to improve understanding of microbiota-brain interactions and biological heterogeneity? Fourth, what microbiome-derived biomarkers and computational prediction models have been developed for disease classification or treatment-response prediction? Fifth, what microbiome-targeted interventions have been evaluated and what is their current level of translational evidence?

### 2.3 Eligibility criteria

Eligibility criteria were developed according to the JBI PCC framework for scoping reviews (17) (Table 1). Studies were considered eligible when they investigated human populations with depression, anxiety disorders, depressive symptoms, anxiety symptoms, or clinically relevant psychiatric phenotypes. Studies involving exclusively animal models, cell culture systems, or non-human experimental models were excluded because the objective of this review was to evaluate translational evidence relevant to human psychiatric healthcare. Regarding the concept of interest, studies were included if they investigated gut microbiome composition, microbial functional characteristics, microbiota-gut-brain interactions, microbial metabolites, multi-omics approaches, microbiome-derived biomarkers, artificial intelligence or machine-learning models using microbiome-related data, or microbiome-targeted interventions. Studies lacking microbiome-related measurements or without direct relevance to psychiatric outcomes were excluded. The contextual scope included clinical cohorts, psychiatric healthcare settings, primary healthcare populations, community-based studies, and population-level investigations conducted worldwide. Particular attention was given to evidence relevant to clinical translation and precision medicine implementation, including emerging research from China. Eligible study designs included observational studies, cohort studies, case-control studies, randomized controlled trials, intervention studies, diagnostic studies, and predictive modelling studies. Reviews, editorials, commentaries, conference abstracts without full-text availability, and study protocols without reported results were excluded. The eligibility criteria were intentionally broad with respect to primary study design to capture the heterogeneous translational evidence characteristic of this emerging field, while restriction to human psychiatric populations and primary empirical studies was applied to maintain direct relevance to clinical precision psychiatry.

**Table 1:** Eligibility criteria according to the PCC framework.

PCC element	Inclusion criteria	Exclusion criteria
Population	Human participants with depression, anxiety disorders, depressive symptoms, anxiety symptoms, stress-related psychiatric phenotypes, or related mental-health outcomes	Animal-only studies, cell culture studies, non-human models
Concept	Studies investigating gut microbiome composition, microbial function, microbiota-gut-brain interactions, metabolomics, lipidomics, multi-omics, biomarkers, artificial intelligence models, or microbiome-targeted interventions	Studies without microbiome-related measurements or without psychiatric relevance
Context	Clinical cohorts, community populations, psychiatric settings, primary healthcare settings, and population-based studies worldwide	Studies unrelated to human healthcare or without relevant clinical context
Study design	Observational studies, cohort studies, case-control studies, randomized controlled trials, intervention studies, diagnostic studies, and predictive modelling studies	Reviews, editorials, commentaries, conference abstracts without full text, protocols without results

### 2.4 Information sources and search strategy

A structured literature search was conducted using PubMed/MEDLINE and PubMed Central from database inception to July 2026, supplemented by Google Scholar and backward citation searching of included studies and relevant reviews. The search strategy was developed according to JBI recommendations for scoping reviews and reported following PRISMA-ScR guidance (17, 18). The search combined controlled vocabulary and free-text terms related to four major concepts: microbiome, psychiatric disorders, precision medicine, and translational technologies. Search terms were adapted according to database requirements and combined using Boolean operators (Table 2).

**Table 2:** Core search strategy.

Concept	Search terms
Microbiome	“microbiome” OR “microbiota” OR “gut microbiome” OR “gut microbiota” OR “intestinal microbiome” OR “microbiota-gut-brain axis”
Psychiatric disorders	“depression” OR “major depressive disorder” OR “depressive disorder” OR “anxiety” OR “generalized anxiety disorder” OR “psychiatric disorder” OR “mental health”
Precision psychiatry	“precision medicine” OR “precision psychiatry” OR “personalized medicine” OR “individualized treatment”
Multi-omics and prediction	“metagenomics” OR “metabolomics” OR “lipidomics” OR “multi-omics” OR “biomarker” OR “machine learning” OR “artificial intelligence” OR “prediction model”

### 2.5 Study selection process

All identified records were imported into reference management software, and duplicate records were removed before screening. The selection process consisted of two sequential stages. During the first stage, titles and abstracts were independently screened by two reviewers against the predefined eligibility criteria. Potentially relevant records were subsequently assessed independently by both reviewers through full-text review to determine final inclusion. To improve consistency, the eligibility criteria were piloted on a subset of retrieved records before formal screening. Disagreements regarding study eligibility were resolved through discussion and consensus, with consultation of an additional reviewer when consensus could not be reached. The study selection process is presented in a PRISMA-ScR flow diagram (Figure 1).

**Figure 1:**
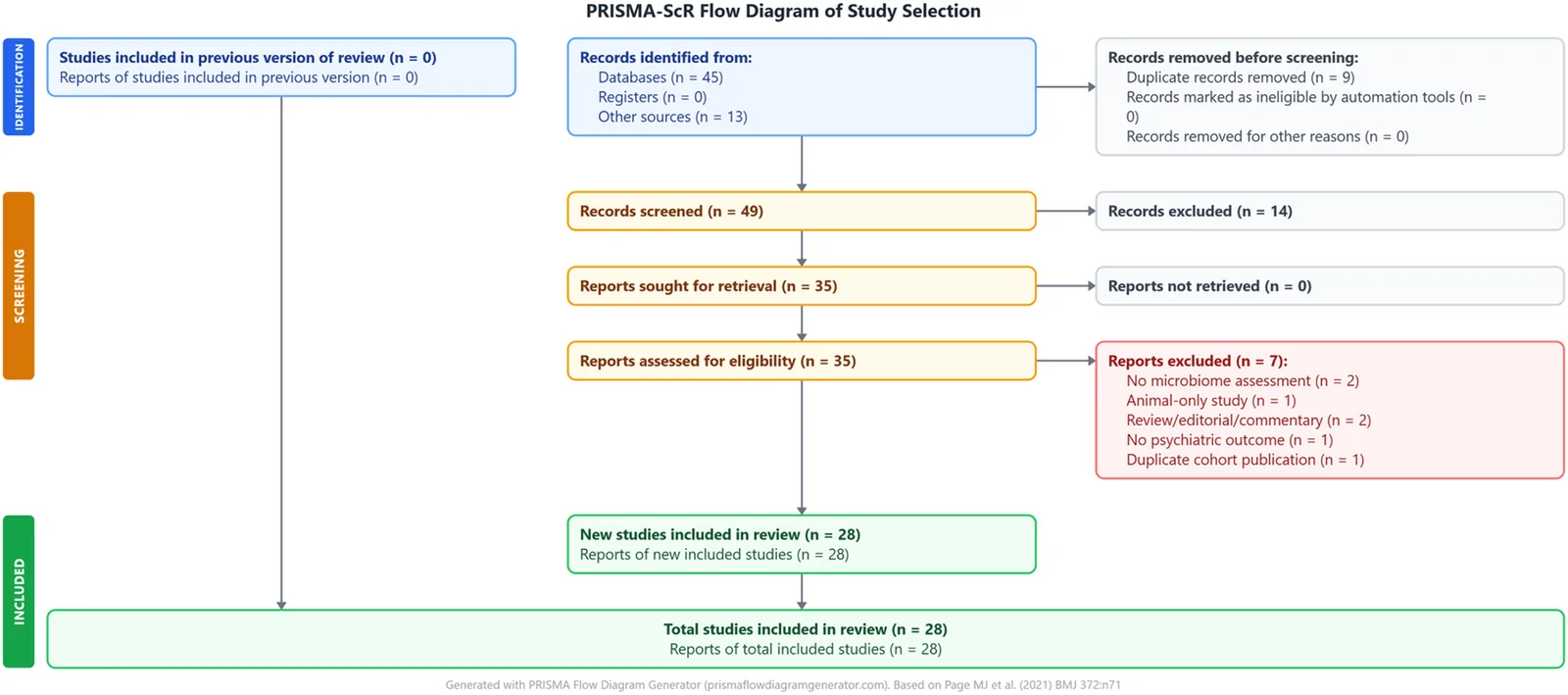
PRISMA-scR flow diagram illustrating the identification, screening, eligibility assessment, and inclusion of studies investigating microbiome-driven precision psychiatry. The selection process was conducted according to the Preferred Reporting Items for Systematic Reviews and Meta-Analyses Extension for Scoping Reviews (PRISMA-ScR) guidelines.

### 2.6 Data extraction and charting

A standardized data-charting form was developed based on JBI recommendations for scoping reviews (17). The extraction framework was designed to capture both descriptive characteristics of included studies and information relevant to the translational potential of microbiome-driven precision psychiatry. Extracted bibliographic information included first author, publication year, journal, and geographic location. Study characteristics included study design, recruitment setting, sample size, participant demographics, and relevant clinical characteristics. Psychiatric-related information included diagnostic criteria, psychiatric phenotype, symptom severity, psychiatric medication exposure, and comorbid conditions when available. Microbiome-related variables included biological sample type, sequencing approach, analytical platform, bioinformatics methodology, and reported microbial signatures. Functional biological information was extracted when available, including microbial metabolic pathways, metabolomics, lipidomics, transcriptomics, immune profiling, and host biological measurements associated with microbiota-brain interactions. Additional clinical translation variables included biomarker development, predictive modelling approaches, artificial intelligence applications, intervention strategies, and clinical outcomes. Potential microbiome confounding factors, including dietary characteristics, antibiotic exposure, lifestyle factors, body mass index, and medication history, were extracted whenever reported because these variables substantially influence microbiome composition and interpretation of psychiatric associations.

### 2.7 Data synthesis and presentation

Evidence synthesis was performed using a narrative descriptive approach consistent with scoping review methodology. Because microbiome-driven precision psychiatry encompasses diverse study designs, analytical platforms, psychiatric phenotypes, and outcome measures, quantitative meta-analysis was not considered appropriate. The extracted evidence was organized into major conceptual domains, including the evolution of microbiome psychiatry, microbial signatures associated with depression and anxiety, functional microbiome-host interactions identified through multi-omics approaches, microbiome-derived biomarkers, artificial intelligence applications, microbiome-targeted interventions, and future clinical translation. Particular emphasis was placed on identifying methodological trends, biological mechanisms, translational opportunities, and knowledge gaps. Findings were interpreted within the broader framework of precision psychiatry, focusing on how microbiome-derived information may complement existing clinical approaches to improve biological stratification and individualized treatment strategies. The formal evidence synthesis was restricted to the primary human studies meeting the predefined eligibility criteria. Narrative reviews, conceptual and methodological articles, and preclinical studies that did not meet these criteria were not included in the charted evidence base or counted among the included studies; where cited, they were used only to provide mechanistic, methodological, or translational context for interpretation of the human evidence.

### 2.8 Methodological considerations

Consistent with the evidence-mapping purpose of this scoping review, a formal study-level risk-of-bias or certainty-of-evidence assessment was not undertaken because the objective was to characterize the breadth, methodological diversity, and translational development of the literature rather than estimate pooled intervention effects or generate formal certainty-of-evidence judgments. Instead, methodological characteristics and limitations relevant to reliability, reproducibility, and clinical translation were considered descriptively throughout the synthesis (17). Evaluation focused on factors affecting reliability, reproducibility, and translational potential, including sample size, recruitment strategy, diagnostic criteria, participant characteristics, medication exposure, dietary influences, and comorbid conditions. Methodological aspects of microbiome analysis were also considered, including sequencing methodology, bioinformatics pipelines, normalization approaches, statistical validation, and reproducibility across populations. For studies involving artificial intelligence or predictive modelling, additional attention was given to model development procedures, sample-to-feature ratios, external validation, interpretability, and potential risks of overfitting.

### 2.9 Protocol and transparency

This review was developed according to JBI methodological guidance and PRISMA-ScR reporting recommendations (17, 18). The review protocol was not prospectively registered in a public registry. The protocol specified the review objectives, eligibility criteria, search strategy, screening procedures, data-charting framework, and synthesis approach.

### 2.10 Ethics statement

This review involves synthesis of previously published literature and does not include recruitment of human participants, experimental interventions, or access to identifiable patient-level information. Therefore, ethical approval was not required. All included studies were considered according to their original ethical approval statements and reporting standards.

## 3 Results

### 3.1 Literature search results and study selection

The literature search identified 58 records from the predefined information sources, including searches of PubMed/MEDLINE and PubMed Central, supplemented by citation searching through Google Scholar and reference lists of relevant publications. After removal of duplicate records, 49 unique records remained for title and abstract screening. During the initial screening phase, 14 records were excluded because they were unrelated to human psychiatric populations, lacked microbiome-based investigations, represented secondary publications, or did not address depression, anxiety, or clinically relevant psychiatric outcomes. A total of 35 full-text articles were assessed for eligibility. Following full-text evaluation, 7 articles were excluded due to absence of direct microbiome measurements (*n* = 2), non-human experimental designs (*n* = 1), review or commentary articles without primary data (*n* = 2), lack of relevant psychiatric outcomes (*n* = 1), or duplicate reporting of an overlapping cohort (*n* = 1). Finally, 28 primary studies were included in the scoping review synthesis (Figure 1). The evidence base was predominantly observational, comprising case-control studies, population-based and clinical cohorts, and prospective studies evaluating treatment response, together with a smaller number of randomized controlled intervention trials. Across these designs, more recent studies incorporated multi-omics profiling and predictive or machine-learning approaches, although these represent overlapping methodological categories rather than mutually exclusive study designs. No animal-only studies were included in the formal scoping-review synthesis; preclinical evidence discussed elsewhere in the manuscript is used only to provide mechanistic context. Collectively, the included studies demonstrated the transition of microbiome psychiatry from early investigations focused on microbial compositional differences toward increasingly integrative approaches incorporating microbial functional pathways, host metabolic interactions, biomarker discovery, computational prediction, and personalized therapeutic strategies.

### 3.2 Characteristics of included studies

Overall, the methodological profile of the evidence base was characterized by a predominance of observational designs, substantial heterogeneity in participant and analytical characteristics, and limited independent validation of emerging predictive approaches. The included studies collectively illustrate the progressive evolution of microbiome psychiatry from initial investigations of microbial composition toward increasingly integrated biological and translational frameworks (Table 3). Consistent with this distribution, observational evidence dominated the literature, while randomized intervention studies remained comparatively limited. Major depressive disorder (MDD) represented the most extensively investigated psychiatric condition, whereas anxiety disorders, particularly generalized anxiety disorder (GAD), constituted a comparatively smaller but rapidly expanding area of research. Early clinical studies primarily focused on identifying differences in gut microbial composition between individuals with psychiatric disorders and healthy controls, whereas more recent investigations have increasingly examined microbial functions, host-microbiome interactions, predictive biomarkers, and individualized therapeutic approaches (9, 11, 19). Detailed study-level characteristics, including study design, country or population, sample size, biological sample type, microbiome methodology, sequencing platform, additional omics analyses, and primary scientific contribution, are presented in Supplementary Table S2, enabling direct methodological comparison across the included evidence base.

**Table 3:** Evolution of microbiome-driven precision psychiatry evidence based on included studies.

Research stage	Representative evidence	Methodological approach	Major contribution	Remaining limitation
Microbial profiling era	Jiang et al. (2015) (9); Jiang et al. (2018) (19)	16S rRNA sequencing	Demonstrated associations between gut microbial composition and depressive/anxiety phenotypes	Limited functional interpretation and reproducibility
Functional microbiome era	Valles-Colomer et al. (2019) (11)	Shotgun metagenomics	Revealed microbial functional capacity and neuroactive pathways associated with mental health outcomes	Limited clinical validation
Microbiome-host interaction era	Wang et al. (2023) (20); Zu et al. (2024) (21)	Microbiome sequencing integrated with metabolomics	Identified microbial-host metabolic networks relevant to psychiatric heterogeneity	Complex biological interpretation
Treatment-response prediction era	Dong et al. (2022) (22); Wang et al. (2023) (20)	Clinical cohorts with predictive modelling	Explored microbiome-informed antidepressant response prediction	Lack of external validation
AI-enabled precision approaches	Emerging machine-learning studies	Computational integration of microbiome and clinical variables	Enabled high-dimensional pattern recognition and biomarker discovery	Small datasets and limited interpretability
Intervention translation era	Romijn et al. (2017) (25)	Randomized controlled trials	Evaluated microbiome modulation as adjunctive psychiatric therapy	Heterogeneous interventions and outcomes

Early human microbiome psychiatry studies predominantly used 16S rRNA sequencing and collectively demonstrated differences in gut microbial community structure across depressive and anxiety phenotypes. However, these findings were largely derived from cross-sectional studies and showed substantial taxonomic heterogeneity, limiting causal inference, mechanistic interpretation, and reproducibility across populations (9, 19). Several included studies were conducted in Asian populations, including cohorts from China investigating depression, anxiety, and treatment-related microbiome characteristics. The geographic settings of the included studies are summarized in Supplementary Table S2. These studies frequently included clinically diagnosed psychiatric populations, including medication-naïve individuals and patients receiving antidepressant treatment, providing valuable insights into microbiome alterations within real-world clinical settings (9, 11, 19). However, considerable heterogeneity existed across studies regarding participant characteristics, psychiatric diagnostic criteria, medication exposure, dietary patterns, geographic background, sequencing platforms, and analytical pipelines. Such variability represents a major challenge for interpretation because gut microbial composition is strongly influenced by host lifestyle, environmental exposures, metabolic status, and pharmacological factors (11, 12).

Methodological approaches evolved substantially across the included literature. While early investigations primarily focused on microbial abundance and diversity using 16S rRNA sequencing, later studies increasingly adopted shotgun metagenomics and functional analyses to investigate microbial metabolic capacity. These advances shifted the field from asking which microorganisms are altered toward understanding which biological functions are affected. Collectively, later metagenomic and functional studies shifted attention from individual taxa toward microbial functional capacity, including neuroactive and metabolic pathways associated with mental-health phenotypes, supporting the broader relevance of functional microbial characteristics over isolated taxonomic signals (11). A defining feature of recent microbiome psychiatry research was the integration of microbial data with host molecular information. Multi-omics investigations combining microbiome sequencing with metabolomics, lipidomics, immune profiling, and other molecular measurements have provided evidence that psychiatric phenotypes may reflect complex interactions among microbial communities, microbial-derived metabolites, and host biological pathways. Recent studies in MDD have identified microbiome-associated metabolic alterations and microbial determinants associated with antidepressant treatment outcomes, supporting a transition toward biologically informed stratification of psychiatric disorders (20, 21). These approaches are particularly relevant for precision psychiatry because they may help identify biologically distinct subgroups within clinically heterogeneous disorders.

An increasing number of studies have explored whether microbiome-derived features can contribute to predicting treatment response. Prospective clinical cohorts have investigated whether baseline microbial composition or microbiome-associated metabolic signatures could differentiate antidepressant responders from non-responders. Collectively, prospective treatment-response studies suggest that baseline microbial composition and microbiome-associated metabolic signatures may differ between antidepressant responders and non-responders, supporting their potential relevance to individualized treatment prediction (20, 22). Although these findings represent important progress toward individualized treatment strategies, current predictive models remain exploratory because most studies have limited sample sizes, lack independent external validation, and require replication across diverse populations. Artificial intelligence (AI) and machine-learning approaches represent a further emerging direction within microbiome psychiatry. A limited but growing number of studies have applied computational approaches to high-dimensional microbiome datasets to identify microbial patterns associated with psychiatric phenotypes, symptom severity, or treatment outcomes. These approaches provide opportunities to integrate microbiome features with clinical and molecular information; however, their clinical translation remains constrained by challenges including small training datasets, overfitting risk, limited biological interpretability, and insufficient validation in independent cohorts. Therefore, AI currently serves primarily as an analytical framework for biomarker discovery rather than an established clinical decision-making tool.

Microbiome-targeted intervention studies represent the initial efforts to translate microbiome psychiatry from observational research into therapeutic applications. Randomized controlled trials investigating probiotics, psychobiotics, and microbiome-modulating strategies have reported potential improvements in depressive symptoms, particularly when used as adjunctive approaches alongside conventional treatment. However, interpretation remains challenging because interventions differ substantially in microbial strains, dosage, treatment duration, participant characteristics, baseline microbiome composition, and outcome measures. Studies evaluating probiotic supplementation have provided preliminary evidence supporting the feasibility of microbiome modulation, while also highlighting the need for larger, standardized, and mechanistically informed clinical trials (23, 24).

Overall, the characteristics of included studies demonstrate a clear transition from descriptive microbiome profiling toward integrated frameworks combining functional microbiology, multi-omics analysis, computational modelling, and individualized interventions. Despite substantial progress, current evidence remains primarily exploratory, and clinically validated microbiome-based diagnostic markers or treatment-selection tools have not yet been established. Future advances will depend on harmonized analytical pipelines, longitudinal multi-omics datasets, rigorous external validation, and incorporation of host-specific and environmental factors to determine whether microbiome-guided approaches can contribute to clinically actionable precision psychiatry. Although the included literature spans several translational domains, some areas of the synthesis remain supported by a relatively limited number of primary studies, and conclusions regarding biomarkers, predictive models, and individualized interventions should therefore be interpreted as evidence mapping rather than confirmation of clinical effectiveness.

### 3.3 Evolution of microbiome psychiatry research: from microbial signatures to precision psychiatry

The field of microbiome psychiatry has undergone a substantial conceptual transformation over the past decade, progressing from initial efforts to identify microbial differences associated with psychiatric disorders toward an integrated systems-level framework incorporating microbial ecology, host biology, computational approaches, and individualized therapeutic strategies. Early investigations primarily addressed whether measurable alterations in gut microbial composition accompanied psychiatric disorders. These pioneering studies established the biological foundation for the microbiota-gut-brain axis by demonstrating that individuals with major depressive disorder (MDD) and anxiety disorders exhibit distinct microbial profiles compared with healthy populations (9, 19). However, the specific taxa reported across studies have shown limited consistency, reflecting both biological and methodological heterogeneity. Geographic and dietary differences, medication exposure, and variation in participant characteristics can shape the underlying gut microbial community, while differences in sequencing methods, bioinformatics processing, and statistical pipelines can influence which microbial features are detected and reported across cohorts (11, 12).

Early microbiome psychiatry was dominated by 16S rRNA-based taxonomic profiling, which collectively established associations between depressive and anxiety phenotypes and altered gut microbial community structure. However, inconsistent taxonomic findings, small cohorts, cross-sectional designs, and limited mechanistic resolution highlighted the limitations of composition-based approaches. The field subsequently shifted toward metagenomic and functional characterization, emphasizing microbial metabolic capacity, neuroactive pathways, and host-microbiome interactions rather than isolated microbial taxa (9, 11, 19).

The emergence of multi-omics technologies represented a further transition from descriptive microbiome profiling toward mechanistic investigation of microbiome-host interactions. Integration of microbiome sequencing with metabolomics, lipidomics, transcriptomics, immune profiling, and clinical phenotyping has enabled researchers to examine how microbial alterations translate into systemic biological changes. Recent multi-omics studies in MDD have identified coordinated alterations involving microbial composition, circulating metabolites, inflammatory pathways, and metabolic networks, suggesting that psychiatric phenotypes may arise from complex disturbances within interconnected host-microbiome systems rather than isolated microbial abnormalities (20, 21). This systems biology perspective provides an opportunity to identify biologically meaningful patient subgroups and move beyond symptom-based classification toward molecularly informed stratification.

A further evolution of the field has been the transition from disease association toward biomarker discovery and treatment-response prediction. Conventional psychiatric diagnoses encompass substantial biological heterogeneity, resulting in variable treatment outcomes among individuals with similar clinical presentations. Microbiome-based approaches aim to complement clinical assessment by identifying molecular signatures associated with disease susceptibility, symptom severity, or therapeutic response. Clinical cohort studies have suggested that baseline microbial composition and microbiome-associated metabolic characteristics may differ between antidepressant responders and non-responders, indicating potential utility for individualized treatment prediction (20, 22). However, current evidence remains preliminary because most predictive models require larger cohorts, independent external validation, and standardized analytical frameworks before clinical implementation.

The integration of artificial intelligence (AI) and machine-learning approaches represents the most recent analytical advancement in microbiome psychiatry. Microbiome datasets contain highly dimensional and complex biological information involving nonlinear relationships among microbial communities, metabolites, host factors, and clinical characteristics. Machine-learning approaches provide new opportunities to identify complex microbial patterns associated with psychiatric phenotypes, symptom severity, and treatment outcomes. Nevertheless, current applications remain primarily exploratory, with major limitations including limited sample sizes, overfitting risk, inconsistent feature-selection strategies, insufficient external validation, and challenges in biological interpretation (20). Therefore, AI currently functions primarily as an enabling framework for biomarker discovery and data integration rather than an established clinical diagnostic technology.

Alongside biomarker development, microbiome psychiatry has entered an early translational phase through microbiome-targeted interventions. Probiotic, prebiotic, and synbiotic strategies have been investigated as potential adjunctive approaches to influence microbial composition, metabolic activity, and host physiological pathways. Randomized controlled trials have reported improvements in depressive symptoms following probiotic supplementation, supporting the biological plausibility of microbiome modulation as a therapeutic strategy (23). Similarly, probiotic add-on interventions have demonstrated potential effects on depressive symptoms and microbiome-associated measures, although interpretation remains limited by heterogeneous microbial formulations, intervention durations, participant characteristics, and clinical outcome assessments (24). At present, microbiome-based interventions should be considered complementary strategies requiring further validation rather than replacements for established psychiatric treatments.

Overall, the evolution of microbiome psychiatry reflects a broader transition in biomedical research from reductionist disease classification toward individualized biological models. The field has progressed through three interconnected stages: identifying microbial signatures associated with psychiatric phenotypes, functionally characterizing microbiome-host interactions through multi-omics approaches, and developing predictive and therapeutic frameworks for precision psychiatry (Figure 2). Despite substantial advances, clinical translation remains limited by the absence of standardized analytical pipelines, insufficient longitudinal datasets, incomplete integration of environmental and host-specific factors, and the need for validation across diverse populations. Future progress will likely depend on combining microbiome information with complementary biological and clinical data, including genomics, metabolomics, immune profiling, digital phenotyping, and longitudinal clinical measurements. Rather than replacing existing psychiatric classification systems, microbiome-based approaches may provide additional biological information that improves patient stratification, supports treatment-response prediction, and facilitates more individualized therapeutic strategies. Thus, microbiome psychiatry is transitioning from an exploratory discipline focused on microbial associations toward an emerging precision medicine framework grounded in systems biology and personalized care.

**Figure 2:**
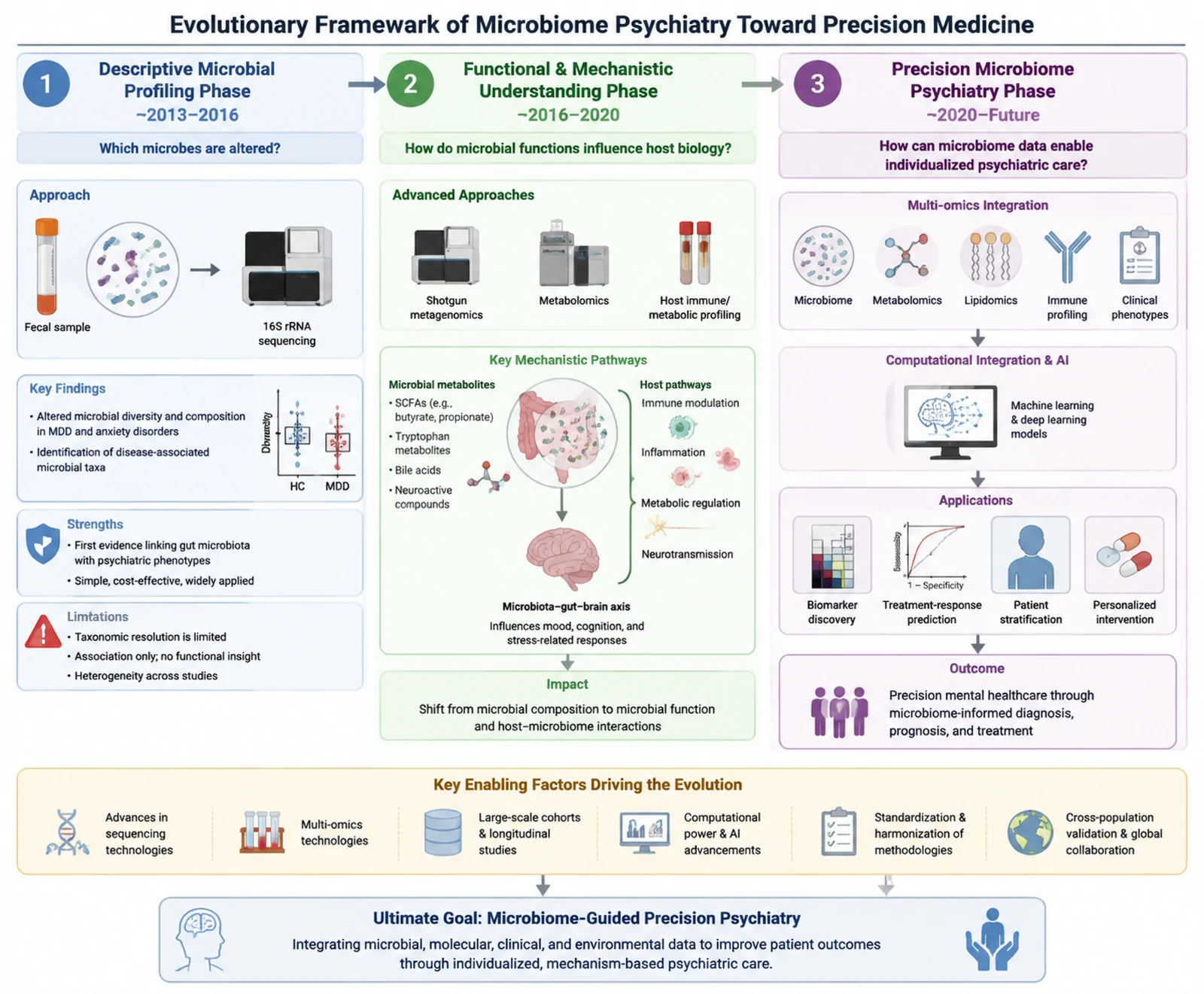
Illustrative conceptual evolution of microbiome psychiatry toward precision medicine. This figure provides a conceptual overview of the broad methodological progression of microbiome psychiatry from early taxonomic characterization toward functional profiling, multi-omics integration, biomarker development, artificial intelligence-assisted analysis, and emerging precision-psychiatry applications. Initial investigations predominantly characterized microbial composition using approaches such as 16S rRNA sequencing, followed by increasing emphasis on microbial functional pathways, metabolites, and host-microbiome interactions. More recent research has incorporated multi-omics profiling, predictive modelling, and individualized therapeutic concepts. The temporal phases shown in the figure are approximate and are intended only to illustrate broad developments in the field; they were not derived from a formal bibliometric analysis or quantitative temporal trend assessment.

### 3.4 Microbiome functional states as emerging precision medicine biomarkers

In Sections 3.4–3.10, mechanistic, conceptual, or methodological references falling outside the predefined eligibility criteria are used only as contextual literature and should be distinguished from the 28 human primary studies included in the formal scoping-review synthesis. The future clinical application of microbiome research is unlikely to depend on identification of universal microbial signatures. Instead, precision medicine approaches increasingly emphasize functional microbiome states defined by interactions among microbial composition, metabolic activity, host immune responses, and clinical characteristics. Such functional states may better capture biological heterogeneity among patients with similar psychiatric diagnoses. Multi-omics integration provides a framework for identifying these biological states by connecting microbial genes with downstream molecular consequences. Metagenomics describes microbial functional potential, whereas metabolomics and lipidomics capture biologically active molecules that directly influence host physiology. Integration with immune profiling and clinical phenotyping may further identify patient subgroups characterized by distinct inflammatory, metabolic, or neurobiological mechanisms. Therefore, microbiome-based precision psychiatry should move beyond the search for disease-specific microbial markers toward development of individualized host-microbiome profiles capable of predicting disease trajectory and therapeutic response.

### 3.5 Microbiome signatures associated with depression: from taxonomic alterations to functional biological pathways

Major depressive disorder (MDD) represents the most extensively investigated psychiatric condition within microbiome research and has provided much of the foundational evidence supporting the microbiota-gut-brain axis concept. Early studies primarily focused on identifying differences in gut microbial composition between individuals with MDD and healthy controls, establishing that depressive phenotypes are associated with measurable alterations in intestinal microbial ecosystems. However, subsequent research has demonstrated that depression-associated microbiome alterations are substantially more complex than changes in individual bacterial taxa. Current evidence indicates that MDD-associated microbial disturbances involve interconnected alterations in community structure, metabolic activity, immune regulation, and microbial-derived signaling pathways rather than a single reproducible microbial signature (9, 11). The initial generation of depression microbiome studies relied predominantly on 16S rRNA sequencing to characterize microbial community composition. Jiang et al. (2015) provided one of the earliest human demonstrations that individuals with MDD exhibit altered fecal microbiota profiles compared with healthy controls, supporting the hypothesis that intestinal microbial ecosystems may participate in biological processes associated with depression. However, because these studies were primarily cross-sectional, microbial alterations could not be clearly distinguished as contributors, consequences, or correlates of depressive illness. Lifestyle factors frequently associated with depression, including dietary changes, reduced physical activity, and medication exposure, represent additional variables that may influence microbial composition and complicate interpretation. Medication exposure is particularly important when interpreting these signatures because microbial differences observed in treated patients may reflect the psychiatric disorder, treatment-related effects on the microbiome, or both. Studies involving medication-naïve participants and longitudinal sampling before and after treatment are therefore particularly valuable for distinguishing disease-associated microbial alterations from treatment-associated changes.

Attempts to identify consistent microbial biomarkers for depression have revealed substantial heterogeneity across populations. Across the included human studies, taxonomic associations were not sufficiently consistent to support designation of a reproducible set of microbial taxa as uniformly increased or decreased in MDD. Recurrently implicated groups, including SCFA-associated members of the Lachnospiraceae and Ruminococcaceae families, were not reported with sufficient consistency across cohorts to establish a uniform direction of association. Differences in the specific taxa detected and their reported direction across studies should therefore be interpreted as evidence of taxonomic heterogeneity rather than as validated disease-specific microbial signatures. Such variability may reflect differences in geography, diet, medication exposure, disease severity, participant characteristics, sequencing resolution, bioinformatics pipelines, and statistical approaches. These inconsistencies suggest that depression is unlikely to be characterized by a universal microbial fingerprint; instead, microbiome alterations may represent context-dependent ecosystem disturbances influenced by host and environmental factors. A major conceptual shift in the field has been the transition from taxonomic profiling toward functional microbiome interpretation. Microbial composition alone provides limited information regarding biological activity, whereas microbial metabolites and functional pathways may more directly influence host physiology. Valles-Colomer et al. (2019) demonstrated that microbial functional capacity, including pathways associated with neuroactive compound production, was associated with depression-related outcomes and quality-of-life measures. These findings supported the view that microbiome contributions to depression may depend more strongly on microbial functions, metabolic outputs, and host interactions than on the presence or abundance of individual bacterial species.

Several biological pathways may explain how microbiome-derived information can contribute to precision stratification of depressive phenotypes (Table 4). Rather than representing a single disease mechanism, microbial alterations may define biologically distinct subgroups characterized by differences in metabolic activity, inflammatory status, and treatment responsiveness. Microbiota-derived metabolites, including SCFAs, tryptophan-related metabolites, and other neuroactive compounds, represent plausible mediators of gut-brain communication, but the level of mechanistic evidence differs substantially between experimental models and human studies. Human investigations primarily demonstrate associations among microbial composition, metabolite profiles, inflammatory markers, and psychiatric phenotypes, whereas direct evidence for SCFA-mediated histone deacetylase inhibition, modulation of microglial activity, and specific neurotransmitter or neuroimmune effects derives largely from experimental and preclinical models (6, 7). Accordingly, these cellular mechanisms should be interpreted as biologically plausible pathways rather than mechanisms conclusively demonstrated in patients with depression. Human studies are currently more informative regarding associations between altered SCFA-producing communities or circulating microbial metabolites and psychiatric phenotypes than regarding direct causal effects within the central nervous system.

**Table 4:** Major microbiome-associated biological pathways implicated in major depressive disorder.

Biological pathway	Microbiome-related alteration	Potential relevance to depression biology	Representative evidence
Microbial ecosystem disruption	Altered microbial community structure and reduced ecosystem stability	May influence gut-brain communication and host metabolic balance	(9)
SCFA-associated pathways	Altered abundance or function of SCFA-producing microbial communities	Regulation of intestinal barrier integrity, inflammation, and neural signalling	(8)
Neuroactive metabolite pathways	Altered microbial pathways involved in neurotransmitter-related metabolism	Potential modulation of CNS signalling pathways	(11)
Immune and inflammatory regulation	Microbiome-associated changes in inflammatory signalling	Potential influence on neuroimmune communication and microglial activity	(7, 26)
Microbiome-metabolome interactions	Altered microbial metabolites and host metabolic networks	Candidate biological stratification markers	(20, 21)
Treatment-response modulation	Baseline microbial patterns associated with antidepressant outcomes	Potential prediction of therapeutic response	(20, 22)

Another important mechanism involves microbiome-mediated regulation of immune and inflammatory processes. A subset of individuals with depression exhibit chronic low-grade inflammatory alterations, and gut microbial disturbances may contribute to immune activation through changes in intestinal permeability, microbial metabolite production, and host immune signalling. These processes may influence circulating inflammatory mediators and neuroimmune communication, including pathways affecting microglial activation and neuronal function. However, current evidence suggests that inflammation-associated microbiome mechanisms likely represent a biological subtype of depression rather than a universal mechanism shared by all patients. Recent multi-omics investigations have further expanded understanding of depression-associated microbiome signatures by integrating microbial composition with host metabolic information. Studies combining microbiome sequencing with metabolomics have identified coordinated alterations involving microbial features, circulating metabolites, and host metabolic pathways, supporting the concept that depression-associated microbiome disturbances occur within broader host-microbial networks. Wang et al. (2023) demonstrated that microbial characteristics and metabolic alterations were associated with antidepressant treatment outcomes, while additional multi-omics analyses have highlighted interactions between microbial ecosystems and systemic biological pathways (20, 21). These findings support a systems biology framework in which microbiome information may contribute to biological stratification of clinically heterogeneous depressive disorders.

The potential clinical application of depression-associated microbiome signatures is increasingly being explored through biomarker discovery and treatment-response prediction. Conventional psychiatric classification relies largely on symptom-based categories that encompass substantial biological heterogeneity. Microbiome-derived signatures may provide complementary biological information to identify patient subgroups with distinct disease mechanisms or differential therapeutic responses. Studies investigating antidepressant outcomes have suggested that baseline microbial characteristics and microbiome-associated metabolic profiles may contribute to prediction of treatment response; however, these approaches remain investigational and require validation in larger, independent cohorts with standardized analytical frameworks (20, 22).

### 3.6 Microbiome signatures associated with anxiety disorders: current evidence, mechanistic insights, and translational challenges

Compared with major depressive disorder (MDD), microbiome research in anxiety disorders remains at an earlier stage of development, with fewer clinical cohorts, limited longitudinal studies, and comparatively less integration of multi-omics approaches. Nevertheless, emerging evidence indicates that anxiety disorders are associated with alterations in gut microbial composition, microbial functional capacity, and microbiota-derived signalling pathways that may influence neurobiological processes involved in stress regulation and emotional behaviour. Current findings support involvement of the microbiota-gut-brain axis in anxiety-related phenotypes; however, anxiety-associated microbiome signatures remain heterogeneous and have not yet achieved sufficient reproducibility for clinical biomarker applications (7, 19) (Table 5). Initial human investigations of anxiety-associated microbiome alterations focused primarily on generalized anxiety disorder (GAD). Jiang et al. (2018) provided early clinical evidence demonstrating that individuals with GAD exhibited distinct fecal microbial profiles compared with healthy controls using 16S rRNA sequencing. The study identified differences in microbial diversity and relative abundance of specific bacterial groups, suggesting that anxiety disorders may involve alterations in intestinal microbial ecosystems. Importantly, these findings extended microbiome psychiatry beyond depression and supported the possibility that different psychiatric phenotypes may be associated with partially overlapping but biologically distinct microbial alterations.

**Table 5:** Current evidence landscape of microbiome research in anxiety disorders.

Evidence domain	Current evidence	Evidence maturity	Major limitation
Microbial composition	Altered gut microbial profiles reported in GAD and anxiety cohorts	Emerging	Limited replication across populations
Microbial diversity	Associations between community structure and anxiety phenotypes reported	Emerging	Strong influence of lifestyle and environmental factors
Neuroactive metabolites	Potential involvement of serotonin-, GABA-, and tryptophan-related microbial pathways	Mechanistically plausible	Limited direct human metabolite validation
SCFA-associated pathways	Possible regulation of barrier integrity, immune signalling, and neuroimmune communication	Developing	Functional metabolite measurements remain limited
Immune regulation	Microbiota may influence inflammatory pathways relevant to anxiety biology	Developing	Biological heterogeneity among patients
Biomarker discovery	Multi-omics and machine-learning approaches emerging	Exploratory	Lack of external validation
Therapeutic modulation	Probiotic and psychobiotic approaches show potential	Preliminary	Strain-specific effects and inconsistent clinical outcomes

Subsequent studies have explored relationships between gut microbial characteristics, anxiety severity, and clinical heterogeneity. Investigations of patients with anxiety disorders have suggested that microbial features may correlate with symptom burden and biological variation among affected individuals. These observations are particularly relevant for precision psychiatry because anxiety disorders represent clinically heterogeneous conditions encompassing diverse symptom profiles, stress responses, and treatment trajectories. However, current evidence remains limited by relatively small cohorts, cross-sectional designs, heterogeneous diagnostic criteria, and inconsistent microbiome analytical approaches. Similar to early depression research, anxiety microbiome studies initially focused primarily on microbial taxonomic composition. Although differences in microbial abundance and community structure have been reported, reproducibility across populations remains challenging. Similarly, the available anxiety literature does not yet identify individual taxa that are reproducibly increased or decreased across independent cohorts; reported abundance differences therefore remain largely study- and population-dependent. Variation in dietary patterns, medication exposure, age, sex, geographical background, comorbid psychiatric conditions, and environmental factors can substantially influence gut microbial composition. Consequently, a universal anxiety-associated microbiome signature has not yet been established. Current research has therefore shifted toward identifying functional microbial characteristics and host-microbiome interactions that may provide more biologically meaningful information than individual microbial taxa alone.

One of the major biological pathways linking the gut microbiome with anxiety involves microbial regulation of neuroactive metabolites and neurotransmitter-associated signalling. Gut microorganisms participate in the metabolism of tryptophan and other precursors involved in serotonin-related pathways, while also contributing to production or modulation of metabolites associated with gamma-aminobutyric acid (GABA), dopamine, and immune signalling. Because serotonergic and GABAergic systems are central regulators of anxiety-related neural circuits, microbiome-mediated regulation of these pathways represents a biologically plausible mechanism through which intestinal microorganisms may influence emotional behaviour (7). However, direct evidence linking specific microbial metabolites with anxiety phenotypes in humans remains limited, and much of the mechanistic understanding continues to rely on experimental models. Short-chain fatty acid (SCFA)-associated pathways represent another important mechanism through which gut microorganisms may influence anxiety-related processes. Microbial-derived SCFAs contribute to maintenance of intestinal barrier integrity, immune regulation, and communication between peripheral immune systems and the central nervous system. Alterations in SCFA-producing microbial communities have been reported in psychiatric conditions and may influence anxiety vulnerability through changes in intestinal permeability, inflammatory signalling, and neuroimmune regulation (8). However, similar to depression research, SCFA-related findings remain heterogeneous, and functional measurements of microbial metabolites are still lacking in many human anxiety cohorts.

The interaction between gut microbiota and immune regulation represents an additional area of investigation in anxiety biology. Chronic stress and anxiety disorders have been associated with altered hypothalamic-pituitary-adrenal (HPA) axis activity, immune signalling, and inflammatory responses. Microbial dysbiosis may influence these processes through alterations in intestinal barrier function, microbiota-derived metabolites, and systemic immune signaling, providing plausible pathways through which gut microbial disturbances may contribute to neuroimmune regulation (6, 7). Nevertheless, inflammatory alterations are biologically heterogeneous among individuals with anxiety disorders, suggesting that microbiome-mediated immune mechanisms may characterize specific biological subgroups rather than represent universal features of anxiety. Compared with depression, fewer anxiety studies have incorporated multi-omics approaches integrating microbiome sequencing with metabolomics, immune profiling, or other host molecular measurements. However, these approaches represent an important future direction because they may identify functional microbial alterations that are not detectable through taxonomic profiling alone. Integration of microbial and host molecular data may ultimately enable identification of biologically distinct anxiety subtypes with different mechanisms, symptom trajectories, or treatment responses (6). The potential application of microbiome-derived biomarkers for anxiety precision psychiatry remains promising but preliminary. Emerging computational approaches, including machine learning and multi-dimensional biological modelling, may provide opportunities to identify anxiety-related microbial patterns and integrate them with clinical characteristics. However, current predictive models remain limited by small sample sizes, insufficient external validation, inconsistent feature selection strategies, and limited biological interpretation. At present, microbiome signatures cannot be used for diagnosis, prognosis, or treatment selection in routine clinical practice.

Microbiome-targeted interventions represent an emerging translational area in anxiety research. Preclinical studies have demonstrated that manipulation of the gut microbiome can influence stress responses, anxiety-like behaviours, and neurochemical pathways. Human intervention studies investigating probiotics and psychobiotic approaches have reported potential anxiolytic effects; however, findings remain inconsistent because of differences in microbial strains, intervention duration, participant characteristics, baseline microbiome composition, and outcome measures (27). Therefore, microbiome modulation should currently be considered an experimental adjunctive strategy requiring further validation rather than an established therapeutic approach for anxiety disorders.

### 3.7 Multi-omics reveals functional microbiome-host interactions in psychiatric disorders: from metabolites to biological subtypes

The transition from microbial community profiling toward integrated multi-omics analysis represents one of the most important conceptual advances in microbiome psychiatry. Early investigations primarily examined whether psychiatric disorders were associated with alterations in bacterial composition; however, microbial taxonomy alone provides limited information regarding microbial activity, metabolic function, and downstream effects on host physiology. Psychiatric disorders, including major depressive disorder (MDD) and anxiety disorders, are biologically heterogeneous conditions involving interactions among immune regulation, metabolism, endocrine signalling, neural circuitry, and environmental influences. Multi-omics approaches provide a framework for integrating microbiome composition with microbial genes, metabolites, host metabolic pathways, immune responses, and clinical phenotypes to understand these complex biological interactions better (6, 7) (Figure 3; Table 6).

**Figure 3:**
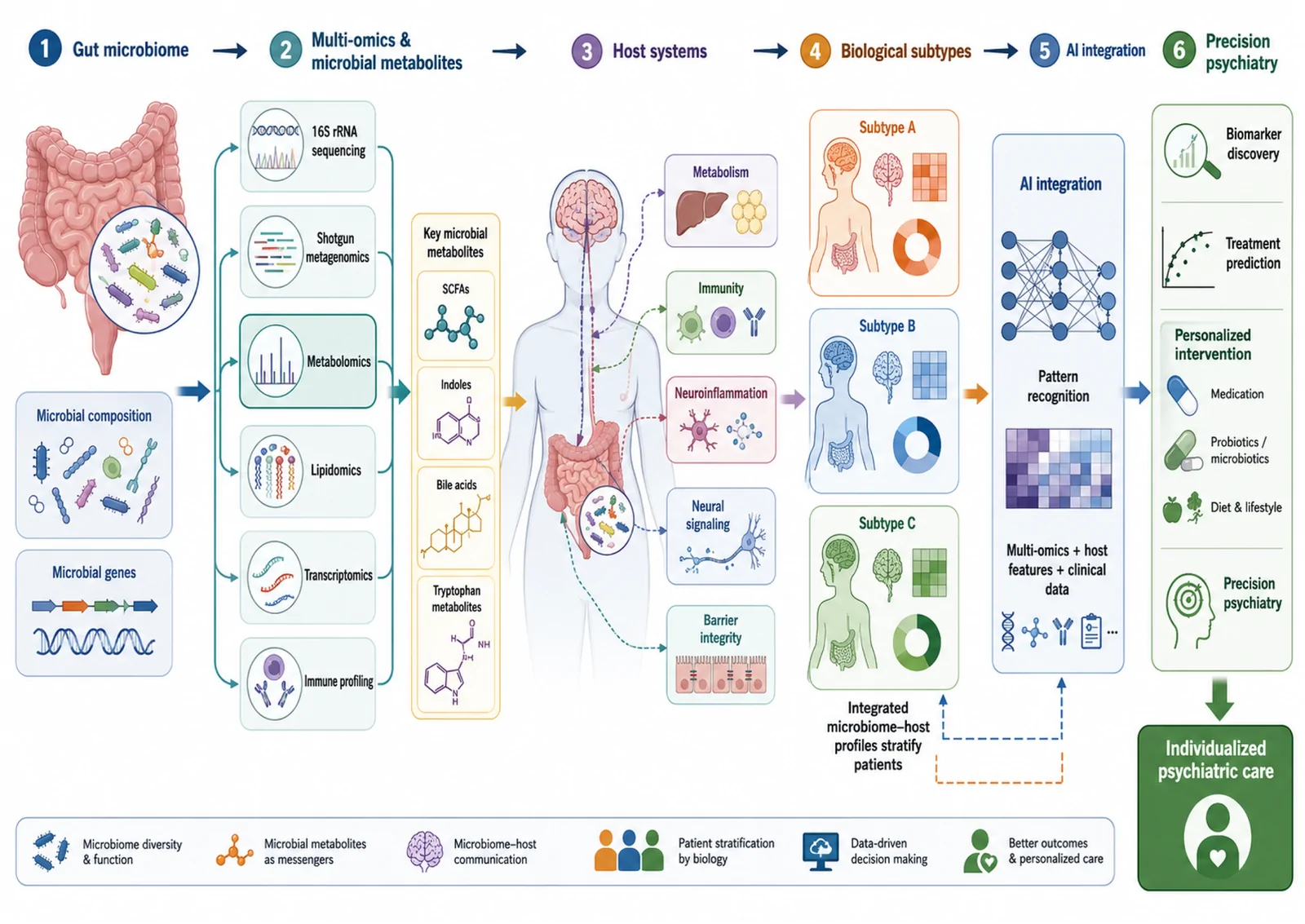
Multi-omics framework linking microbiome-host interactions to precision psychiatry. The integration of multi-omics approaches provides a systems-level framework for understanding how gut microbial alterations contribute to psychiatric heterogeneity beyond taxonomic differences alone. Microbial composition and functional capacity are characterized through approaches including 16S rRNA sequencing, shotgun metagenomics, and other molecular profiling strategies, while microbial-derived metabolites such as short-chain fatty acids (SCFAs), indole derivatives, bile acid metabolites, and tryptophan-related compounds serve as potential mediators of microbiota-host communication. These microbial signals interact with host biological systems, including metabolism, immune regulation, neuroinflammatory pathways, intestinal barrier integrity, and neural signaling. Integration of microbiome, metabolomic, lipidomic, transcriptomic, immune, and clinical datasets enable identification of biologically distinct patient subtypes. Artificial intelligence and machine-learning approaches provide analytical frameworks for integrating complex multidimensional datasets, facilitating biomarker discovery, treatment-response prediction, and development of individualized psychiatric intervention strategies. This framework illustrates the emerging transition from microbiome association studies toward microbiome-informed precision psychiatry.

**Table 6:** Multi-omics approaches enabling microbiome-based precision psychiatry.

Omics layer	Biological information obtained	Contribution to psychiatric research	Current evidence status	Key limitations
16S rRNA sequencing	Microbial composition and diversity	Identification of psychiatric-associated microbial patterns	Established	Limited species/strain-level resolution; provides limited direct information on microbial functional capacity; results are influenced by primer selection, sequencing region, and bioinformatics pipeline
Shotgun metagenomics	Microbial genes and functional pathways	Functional interpretation of microbial activity	Developing	Higher cost and computational requirements; dependence on reference databases and bioinformatics pipelines; functional potential does not necessarily indicate active biological function
Metabolomics	Microbial and host-derived metabolites	Identification of biological mediators linking microbiota and brain function	Emerging-strong	Metabolite identification and annotation remain challenging; microbial and host origins may be difficult to distinguish; profiles are highly sensitive to diet, medications, sample handling, and analytical platform
Lipidomics	Bioactive lipid networks	Investigation of neuroinflammation, membrane biology, and metabolic regulation	Emerging	High molecular complexity, incomplete lipid annotation, platform-dependent coverage, and difficulty distinguishing microbiome-related changes from host metabolic effects
Transcriptomics	Host gene-expression responses	Identification of host pathways affected by microbiome signals	Developing	Strong tissue and temporal dependence, RNA instability, substantial biological variability, and limited ability to establish whether transcriptional changes are directly microbiome-driven
Immune profiling	Cytokines and immune activity	Characterization of microbiome-immune-brain interactions	Developing	Immune markers are dynamic and often nonspecific; results may be influenced by infection, medication, metabolic status, circadian variation, and other inflammatory conditions
Artificial intelligence integration	Multidimensional pattern recognition	Biological subtype discovery and treatment prediction	Exploratory	Risk of overfitting in small high-dimensional datasets, limited interpretability, unstable feature selection, dataset dependence, and insufficient independent external validation

The central contribution of multi-omics microbiome research is the ability to move beyond the question of which microorganisms are altered toward determining which biological functions are disrupted and how these changes influence host systems. Gut microorganisms generate and transform diverse bioactive molecules, including short-chain fatty acids (SCFAs), bile acid derivatives, indole metabolites, and neurotransmitter-related compounds. These microbial products may influence intestinal barrier integrity, immune regulation, metabolic homeostasis, and brain signalling pathways. Therefore, psychiatric phenotypes are increasingly viewed not as consequences of isolated microbial changes but as potential outcomes of complex microbiome-host interaction networks (8).

Metabolomics has been one of the most informative approaches for identifying potential mediators of microbiota-brain communication. Unlike taxonomic profiling, which describes microbial composition, metabolomic analysis provides direct information regarding biologically active molecules that may affect host physiology. Beyond psychiatric cohorts, multi-omics studies in other systemic disease models have similarly demonstrated how microbiome-associated metabolic disturbances can propagate across distal organ axes, including gut-lung interactions following environmental toxicant exposure and gut-serum-multi-organ metabolic and redox networks during aging (28, 29). Experimental traumatic brain injury research has used metabolomics to characterize molecular changes associated with neurogenesis and axonal regeneration (30), while recent work in atrial fibrillation demonstrates that a gut microbiota-derived metabolite can influence distal host signaling and inflammatory cell-death pathways (31). These studies are cited as mechanistic and methodological context and are not part of formal human psychiatric evidence synthesis. SCFAs produced through microbial fermentation of dietary fibres represent one extensively studied pathway because they contribute to intestinal epithelial integrity, immune regulation, microglial activity, and epigenetic regulation through mechanisms including histone deacetylase inhibition (8). However, variability across human studies indicates that metabolite-associated mechanisms are likely context-dependent rather than universal features of psychiatric disorders.

Tryptophan metabolism represents another important microbiome-host signaling pathway relevant to psychiatric biology. Gut microorganisms regulate multiple branches of tryptophan metabolism, including serotonin-related pathways, kynurenine metabolism, and production of indole derivatives. These pathways are particularly relevant because alterations in serotonin availability, immune activation, and neuroactive metabolites have been implicated in depression and anxiety pathophysiology. Multi-omics strategies provide an opportunity to determine whether microbial alterations influence psychiatric phenotypes through specific metabolic pathways rather than nonspecific microbial imbalance (32). Bile acid metabolism has also emerged as an important but relatively underexplored pathway in microbiome psychiatry. Gut microorganisms regulate the transformation of primary bile acids into secondary bile acids through enzymatic modification processes. These metabolites function as signalling molecules through receptors including farnesoid X receptor (FXR) and Takeda G-protein receptor 5 (TGR5), influencing metabolic regulation, immune responses, and potentially neural function. Although bile acid pathways have been extensively investigated in metabolic and inflammatory disorders, emerging psychiatric microbiome research suggests that altered microbial bile acid metabolism may represent another mechanism linking intestinal microbial ecosystems with brain physiology (33).

Recent human studies integrating microbiome sequencing with metabolomics have demonstrated the potential value of multi-dimensional biological profiling for understanding psychiatric heterogeneity. Wang et al. (2023) showed that combining gut microbiome profiles with metabolomic information provided insights into microbial determinants associated with antidepressant treatment outcomes in individuals with MDD. Rather than relying solely on microbial abundance patterns, this approach identified coordinated microbial and metabolic signatures associated with therapeutic response, highlighting the potential of multi-omics information for treatment stratification. Similarly, multi-omics investigations have suggested that MDD may involve coordinated disturbances across microbial ecosystems and host metabolic networks. Zu et al. (2024) reported distinct microbiome-associated metabolic profiles in individuals with MDD, supporting the concept that psychiatric disorders may contain biologically distinct subgroups characterized by different microbiome-metabolic configurations. Such findings are particularly relevant for precision psychiatry because they suggest that clinically similar patients may exhibit different underlying biological mechanisms requiring different therapeutic approaches.

Beyond metabolomics, integration of lipidomics represents an emerging direction for microbiome psychiatry. Lipid molecules regulate neuronal membrane structure, synaptic signalling, inflammatory processes, and oxidative stress responses. Microbiome-associated alterations in lipid metabolism may therefore influence psychiatric phenotypes through effects on neuroinflammation, energy metabolism, and neuronal plasticity. Although lipidomics remains less extensively investigated than metabolomics, its incorporation into future multi-omics frameworks may provide additional resolution for identifying biologically meaningful psychiatric subtypes. Transcriptomic and immune profiling approaches provide complementary information by revealing how microbiome-associated signals influence host cellular responses. Psychiatric disorders involving immune dysregulation may be associated with altered cytokine signalling, immune-cell activity, and neuroimmune communication. Integration of microbiome data with host gene expression and immune profiles may therefore enable identification of patient subsets characterized by distinct inflammatory or metabolic states, addressing the biological heterogeneity that remains unresolved by symptom-based psychiatric classifications.

A major translational implication of multi-omics microbiome research is the possibility of redefining psychiatric disorders according to biological mechanisms rather than clinical symptoms alone. Disorders such as MDD and anxiety encompass diverse clinical presentations and variable treatment responses. Integration of microbial composition, metabolite profiles, inflammatory markers, and host molecular characteristics may allow identification of biologically defined subgroups with improved prediction of disease trajectory and therapeutic response. This represents a critical step toward microbiome-informed precision psychiatry (Wang et al., 2023). Artificial intelligence and machine-learning approaches are increasingly being incorporated into multi-omics microbiome research because of the complexity and dimensionality of integrated datasets. Computational models can identify nonlinear relationships among microbial features, metabolites, and clinical outcomes that conventional statistical methods may not capture. However, current AI-based models remain primarily exploration due to limitations including small datasets, insufficient external validation, and variation in analytical pipelines. Therefore, AI should currently be considered an enabling framework for biological discovery rather than a clinically validated decision-support system.

Despite substantial progress, several challenges continue to limit translation of microbiome multi-omics research into clinical psychiatry. Microbiome profiles are dynamic and influenced by diet, medication exposure, lifestyle, geography, and host genetics. Furthermore, many studies remain cross-sectional, limiting determination of whether microbiome alterations contribute to psychiatric disease development or reflect consequences of illness. Standardized approaches for microbiome sampling, sequencing, metabolomic analysis, and computational integration remain essential for improving reproducibility. Large longitudinal cohorts incorporating microbiome, multi-omics, clinical, and environmental information will be required before microbiome-based biological subtyping can be implemented clinically.

### 3.8 Microbiome-derived biomarkers, artificial intelligence, and treatment prediction: toward individualized psychiatric care

The clinical translation of microbiome psychiatry is increasingly shifting from descriptive characterization of disease-associated microbial alterations toward the development of biologically informed tools for patient stratification and treatment optimization. Conventional psychiatric classification systems rely predominantly on symptom-based criteria and provide limited information regarding underlying biological heterogeneity. Disorders such as major depressive disorder (MDD) and anxiety disorders encompass diverse molecular, metabolic, inflammatory, and neurobiological states, contributing to substantial variability in disease trajectory and therapeutic response. In this context, microbiome-derived biomarkers represent a promising approach for complementing clinical phenotyping by providing measurable biological information associated with disease susceptibility, symptom severity, and treatment outcomes (11, 20) (Table 7). The concept of microbiome biomarkers in psychiatry has evolved considerably. Early investigations primarily focused on identifying individual microbial taxa associated with psychiatric disorders; however, inconsistent replication across populations has demonstrated that single bacterial species are unlikely to represent universal biomarkers. Gut microbial ecosystems are dynamic and influenced by multiple host and environmental factors, including diet, geography, medication exposure, lifestyle, and host genetics. Consequently, current research has shifted toward composite biomarkers integrating microbial community structure, functional pathways, microbial metabolites, and host biological responses rather than relying on isolated taxonomic signatures (6, 7).

**Table 7:** Current status of microbiome-derived biomarkers in precision psychiatry.

Biomarker category	Biological information	Potential application	Evidence maturity
Taxonomic microbial signatures	Relative abundance and community composition	Identification of disease-associated microbial patterns	Early
Functional microbial pathways	Microbial genes and metabolic capacity	Biological pathway-based stratification	Developing
Microbial metabolites	SCFAs, tryptophan metabolites, bile acid derivatives	Identification of biologically distinct subgroups	Emerging
Microbiome-metabolome signatures	Integrated microbial and host metabolic profiles	Treatment-response prediction	Emerging
Machine-learning microbial classifiers	Computational integration of microbial features	Predictive modelling and biomarker discovery	Exploratory
Longitudinal microbiome trajectories	Dynamic microbial changes over time	Disease monitoring and outcome prediction	Early

One of the most clinically relevant applications of microbiome biomarkers is prediction of antidepressant treatment response. Although antidepressant medications remain central to depression management, substantial variability exists in treatment outcomes, and reliable biological tools for guiding therapeutic selection remain unavailable. Microbiome-based approaches have therefore attracted attention because gut microorganisms can influence drug metabolism, immune regulation, neurotransmitter-related pathways, and systemic metabolic processes that may affect treatment response. Several human studies have provided preliminary evidence supporting microbiome-informed treatment prediction. Dong et al. (2022) investigated whether gut microbiome characteristics could serve as indicators of antidepressant treatment outcomes in individuals with MDD and reported associations between microbial profiles, functional pathways, and therapeutic response. Similarly, Wang et al. (2023) integrated microbiome sequencing with metabolomic analysis to identify microbial-metabolic features associated with antidepressant outcomes. These findings suggest that treatment response may be influenced not only by microbial composition but also by broader microbiome-host metabolic interactions, supporting the development of multidimensional predictive frameworks for precision psychiatry.

Beyond treatment prediction, microbiome-derived biomarkers may contribute to biological subtyping of psychiatric disorders. Clinical diagnoses such as MDD encompass patients with diverse biological mechanisms, including inflammatory, metabolic, stress-related, and neurobiological subtypes. Multi-omics microbiome studies suggest that some individuals may exhibit distinct microbial-metabolic profiles associated with specific biological pathways. These findings support the possibility that microbiome information could help identify biologically meaningful patient subgroups requiring different therapeutic strategies (11, 20).

Artificial intelligence (AI) and machine-learning approaches have emerged as important analytical tools for interpreting microbiome-derived biomarker datasets (Figure 4). Microbiome datasets contain highly dimensional information involving microbial features, metabolites, clinical variables, and host characteristics, with complex nonlinear relationships that conventional statistical approaches may not adequately capture. Machine-learning algorithms, including random forests, support vector machines, gradient boosting models, and neural network approaches, provide opportunities to identify predictive patterns associated with psychiatric phenotypes and treatment outcomes. Broader computational and translational research provides additional methodological examples relevant to this progression. Network-based multi-biomarker approaches such as NetAUC illustrate strategies for identifying multivariable biomarker panels in high-dimensional biomedical data (34). Likewise, computational modelling of basal-ganglia dynamics in Parkinson's disease illustrates how complex biological signals can be linked to potential intervention targets (35), while integrative genomic and bioinformatic discovery followed by clinical validation of candidate allergens demonstrates the importance of moving from computational identification toward empirical validation (36). These examples provide methodological context rather than direct evidence for psychiatric microbiome biomarkers. Recent studies have applied machine-learning approaches to microbiome datasets for psychiatric classification and biomarker discovery. Metagenomic investigations have demonstrated that combinations of microbial features may distinguish individuals with MDD from healthy controls, suggesting that microbial networks may contain clinically relevant biological information beyond individual taxa. Other computational studies have explored microbial characteristics associated with symptom severity, treatment resistance, and antidepressant response, highlighting the potential contribution of AI-assisted microbiome analysis to individualized psychiatric care (37).

**Figure 4:**
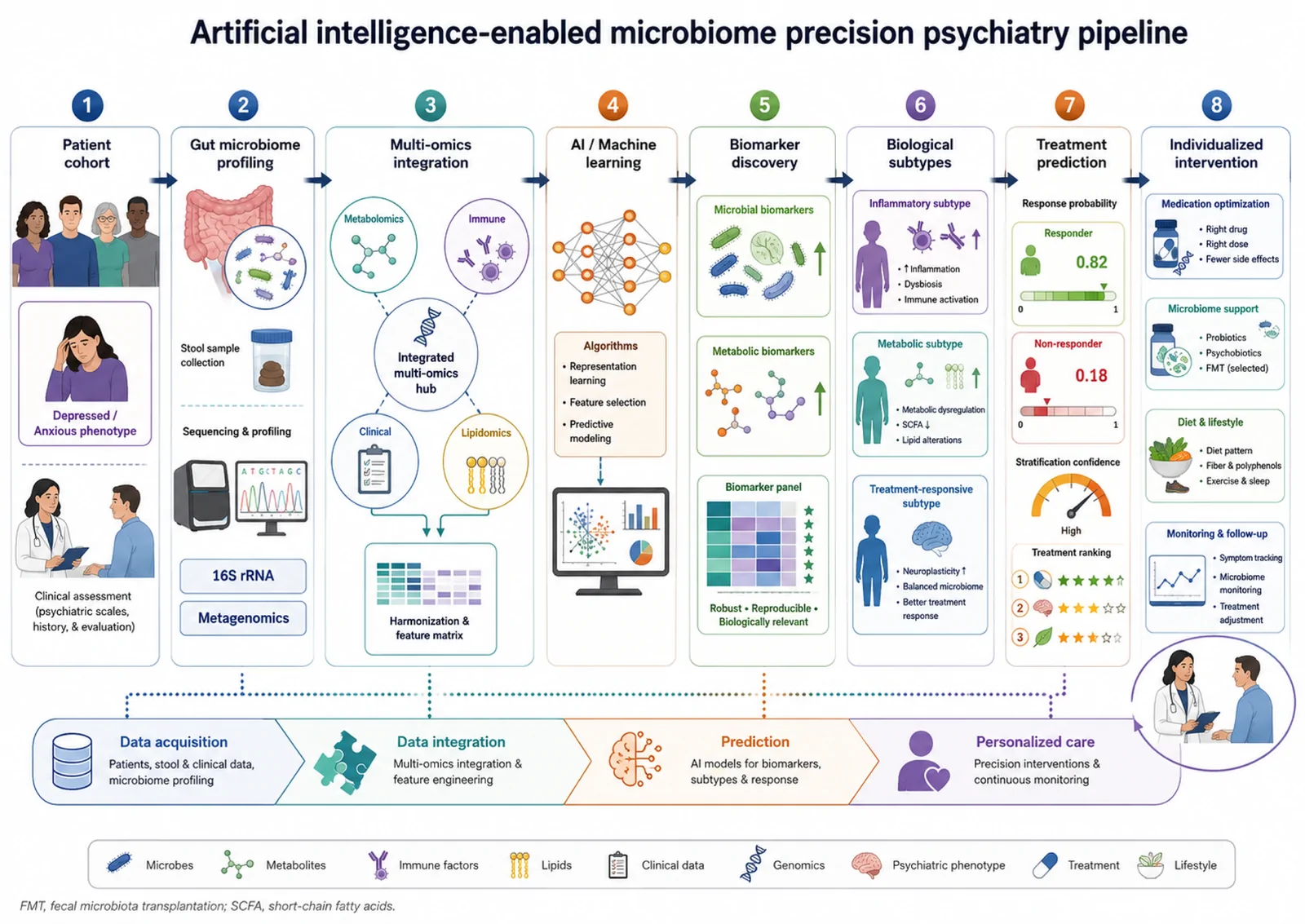
Conceptual framework for AI-enabled microbiome precision psychiatry. This figure illustrates a prospective conceptual framework for integrating microbiome profiling, multi-omics technologies, clinical information, and artificial intelligence in precision psychiatry. Clinical phenotyping and microbiome profiling may be combined with metabolomic, lipidomic, immune, genomic, and other biological data to generate multidimensional patient profiles. Artificial intelligence and machine-learning approaches may support feature integration, pattern recognition, biomarker discovery, and exploratory prediction of psychiatric phenotypes or treatment outcomes. Such approaches could ultimately contribute to biological stratification and individualized intervention strategies. This framework represents a future translational model rather than a validated clinical workflow; current microbiome-derived biomarkers and AI-based predictive models require substantial independent and prospective validation before routine psychiatric implementation.

Despite considerable interest, several limitations currently prevent microbiome-based AI models from clinical implementation. Many studies rely on relatively small cohorts compared with the dimensionality of microbiome datasets, increasing the risk of overfitting and unstable predictions. Furthermore, external validation across independent populations remains limited, and predictive performance frequently declines when models are transferred beyond the original study cohort. The biological interpretation of AI-generated predictions also remains challenging because many computational models function as complex “black boxes.” Future clinical applications will require transparent modelling strategies, standardized data-processing pipelines, and integration with established clinical parameters. An important distinction in microbiome biomarker development is the difference between diagnostic association and clinical utility. A microbial signature capable of distinguishing individuals with depression from healthy controls does not necessarily provide actionable information for patient management. Clinically meaningful biomarkers must demonstrate the ability to improve diagnosis, predict disease trajectory, identify treatment response, or guide therapeutic selection. Accordingly, most microbiome-derived biomarkers identified to date should be considered exploratory candidates derived from discovery cohorts. Evidence of reproducible performance in independent populations remains limited, and the current literature does not yet support microbiome biomarkers with sufficiently consistent external replication for routine diagnostic, prognostic, or treatment-selection use.

Integration of microbiome biomarkers with additional biological and clinical data streams may represent the most realistic pathway toward translation. Combining microbiome profiles with genomics, metabolomics, inflammatory markers, neuroimaging data, electronic health records, and digital behavioural measures may enable development of multidimensional predictive models capable of capturing the biological complexity of psychiatric disorders. Such integrated approaches recognize the microbiome as one component of a broader biological ecosystem rather than an isolated determinant of disease. Future progress in microbiome-informed precision psychiatry will depend on large-scale longitudinal studies that evaluate individuals across disease development, treatment exposure, and clinical outcomes. These studies should incorporate repeated microbiome measurements, dietary information, medication exposure, environmental factors, and multi-omics profiling to determine whether microbiome alterations represent causal contributors, consequences of psychiatric illness, or dynamic biomarkers of treatment response.

### 3.9 Microbiome-targeted interventions and clinical translation: from probiotics to personalized therapeutics

The ultimate goal of microbiome psychiatry is not only to identify disease-associated microbial alterations but also to translate this biological knowledge into effective and individualized therapeutic strategies. As understanding of the microbiota-gut-brain axis has advanced, research has increasingly shifted from observational characterization toward microbiome-targeted interventions designed to modify microbial composition, enhance beneficial microbial functions, regulate host metabolism, and improve psychiatric outcomes. Current and emerging therapeutic approaches include probiotics, prebiotics, synbiotics, dietary modulation, microbial metabolite targeting, and personalized microbiome-based interventions (Table 8). However, despite encouraging experimental and early clinical findings, microbiome-directed therapies remain at an investigational stage and require further validation before routine implementation in psychiatric practice.

**Table 8:** Current and emerging microbiome-targeted therapeutic strategies in precision psychiatry.

Intervention strategy	Proposed mechanism	Current evidence	Major translational challenge
Probiotics	Introduce selected microbial strains and modify microbial metabolites	Randomized trials suggest potential improvement in depressive symptoms	Strain specificity and inconsistent outcomes
Prebiotics	Promote beneficial indigenous microbial growth	Preliminary evidence	Optimal formulation and patient selection
Synbiotics	Combine probiotic organisms with supportive substrates	Emerging evidence	Standardization of combinations
Dietary modulation	Alters microbial ecology and metabolite production	Increasing clinical interest	Separating dietary effects from lifestyle factors
Microbial metabolite targeting	Modifies functional mediators such as SCFAs and other metabolites	Mechanistically promising	Identification of precise therapeutic targets
FMT	Transfers complex microbial ecosystems	Experimental psychiatric evidence	Safety, regulation, donor selection
Personalized microbiome therapy	Multi-omics-guided individualized intervention	Future precision approach	Need for validated prediction models

Probiotics represent the most extensively studied microbiome-targeted intervention in psychiatry. These live microorganisms are proposed to influence mental health through multiple mechanisms, including restoration of microbial balance, enhancement of intestinal barrier integrity, modulation of immune responses, and regulation of microbial metabolite production. Specific probiotic strains, particularly members of the *Lactobacillus* and *Bifidobacterium* genera, have demonstrated potential effects on stress-related behaviour, inflammatory pathways, and neurotransmitter-associated mechanisms in preclinical models (27). In humans, randomized controlled trials have investigated whether probiotic supplementation can reduce depressive and anxiety symptoms either as monotherapy or as an adjunctive strategy alongside conventional psychiatric treatment.

One of the important clinical trials evaluating microbiome modulation in depression was conducted by Kazemi et al. (2019), who performed a randomized controlled trial investigating probiotic and prebiotic supplementation in individuals with major depressive disorder (MDD). The study reported improvements in depressive symptoms and metabolic parameters among participants receiving microbiome-targeted supplementation compared with placebo, suggesting that microbial modulation may influence psychiatric outcomes through combined psychological and metabolic mechanisms (23). However, interpretation of these findings requires caution because intervention duration, microbial strains, baseline microbiome composition, and patient characteristics vary substantially among clinical studies.

More recent trials have attempted to strengthen mechanistic interpretation by integrating microbiome analysis with clinical and biological outcomes. A randomized controlled study evaluating probiotic add-on therapy in depressed patients demonstrated potential effects on depressive symptoms, gut microbial characteristics, and neural-related outcomes, supporting the hypothesis that microbiome modulation may influence psychiatric health through multiple biological pathways (24). These findings represent an important transition from simple symptom-based evaluation toward biologically informed intervention studies; however, larger multicentre trials are required to determine whether microbiome changes are responsible for clinical improvement or associated with therapeutic response.

Beyond probiotics, dietary interventions represent another important strategy for modifying the gut microbiome. Dietary patterns strongly influence microbial composition and metabolic activity, particularly through regulation of fibre availability, SCFA production, and microbial diversity. Diets rich in plant-based fibres, polyphenols, and fermented foods may promote beneficial microbial functions, whereas highly processed dietary patterns may contribute to microbial imbalance and inflammatory signalling (7). Although dietary interventions have shown promise in improving mental health outcomes, separating the direct effects of dietary modification from broader lifestyle changes remains challenging. Future studies integrating dietary assessment with microbiome profiling will be essential for developing personalized nutritional strategies for psychiatric disorders.

Contextual evidence from other disease and experimental systems further illustrates that nutrition-related interventions can interact with intestinal microbial, immune, barrier, and neural processes. Research in type 2 diabetes has described relationships among microbiota modulation, intestinal barrier function, and immunity (38), while broader neurobiological literature highlights nutrient-glial interactions as potential components of precision nutritional intervention (39). In a non-mammalian experimental model, dietary fucoidan has also been associated with changes in gut health, antimicrobial immune responses, and microbiota-gut-brain-axis signaling (40). These studies provide broader mechanistic context and should not be interpreted as evidence of therapeutic efficacy in psychiatric populations.

Prebiotics and synbiotics represent additional approaches aimed at modifying microbial ecosystems indirectly. Prebiotics provide substrates that selectively promote beneficial microbial growth, while synbiotics combine prebiotics with specific probiotic organisms to enhance colonization and metabolic activity. These approaches may offer advantages over single-strain probiotic supplementation because they target microbial community function rather than introducing isolated organisms. Nevertheless, clinical evidence remains limited, and optimal microbial combinations, dosing strategies, treatment duration, and patient selection criteria remain undefined.

An emerging concept in microbiome therapeutics is the development of precision microbiome interventions tailored to individual biological characteristics. Current probiotic strategies generally use standardized microbial strains without considering baseline microbiome composition. However, microbiome psychiatry increasingly recognizes that patients may exhibit distinct microbial ecosystems requiring individualized approaches. Multi-omics profiling may enable identification of patients most likely to benefit from specific interventions by characterizing microbial deficiencies, metabolic abnormalities, and host response patterns (20). For example, individuals with reduced SCFA-producing capacity may theoretically benefit from interventions designed to enhance butyrate production, whereas patients with specific metabolic or inflammatory signatures may require alternative strategies.

Fecal microbiota transplantation (FMT) represents a more intensive microbiome-modifying approach that has demonstrated clinical efficacy in selected gastrointestinal disorders, while its application in psychiatry remains experimental. Interest in neuropsychiatric applications has been stimulated partly by preclinical evidence showing that microbiota transfer can influence behavioral, immune, and metabolic pathways (41). This study is cited solely as mechanistic context outside the formal human evidence synthesis and was not included among the primary studies meeting the review eligibility criteria; its findings should not be interpreted as evidence of established therapeutic efficacy in humans. Translation of FMT to psychiatric disorders raises several additional challenges. Donor selection requires rigorous screening to minimize transmission of infectious agents and other potentially undesirable microbial characteristics, while standardized procedures for donor eligibility, microbiota preparation, administration, and traceability are necessary for reproducible clinical evaluation. Safety assessment should extend beyond immediate adverse events because the durability and long-term biological consequences of modifying a recipient's microbial ecosystem remain uncertain. Future psychiatric FMT trials should therefore incorporate longitudinal monitoring of clinical outcomes, microbiome composition, metabolic responses, and delayed adverse events. Regulatory requirements are also likely to vary across jurisdictions, and psychiatric indications will require appropriate regulatory and ethical oversight before routine clinical use. From an ethical perspective, participants should receive clear information regarding the experimental status of FMT, uncertain psychiatric benefit, potential risks, and the possibility of persistent microbiome alterations. Until efficacy, donor-selection standards, long-term safety, and regulatory pathways are more clearly established, FMT should remain a carefully controlled investigational approach rather than routine treatment for psychiatric disorders.

Another emerging therapeutic direction involves targeting microbiome-derived metabolites rather than microorganisms themselves. Because microbial metabolites represent functional mediators of microbiota-brain communication, manipulating specific metabolic pathways may provide more precise therapeutic opportunities. Potential targets include SCFAs, tryptophan metabolites, bile acid derivatives, and inflammatory mediators. Compared with broad microbiome manipulation, metabolite-based interventions may offer greater specificity by directly modifying biologically active molecules involved in psychiatric pathways. However, the complexity of microbial metabolism and interindividual variability remain major challenges.

The integration of artificial intelligence (AI), multi-omics profiling, and longitudinal clinical monitoring may ultimately enable individualized microbiome therapeutics (Figure 5). Future precision microbiome medicine may involve comprehensive patient profiling that incorporates gut microbiome composition, microbial metabolites, host genetics, immune status, dietary patterns, medication exposure, and clinical characteristics. Machine-learning models could then identify which patients are most likely to benefit from specific interventions and monitor biological responses over time. Such an approach would transform microbiome therapeutics from generalized supplementation strategies toward personalized biological interventions.

**Figure 5:**
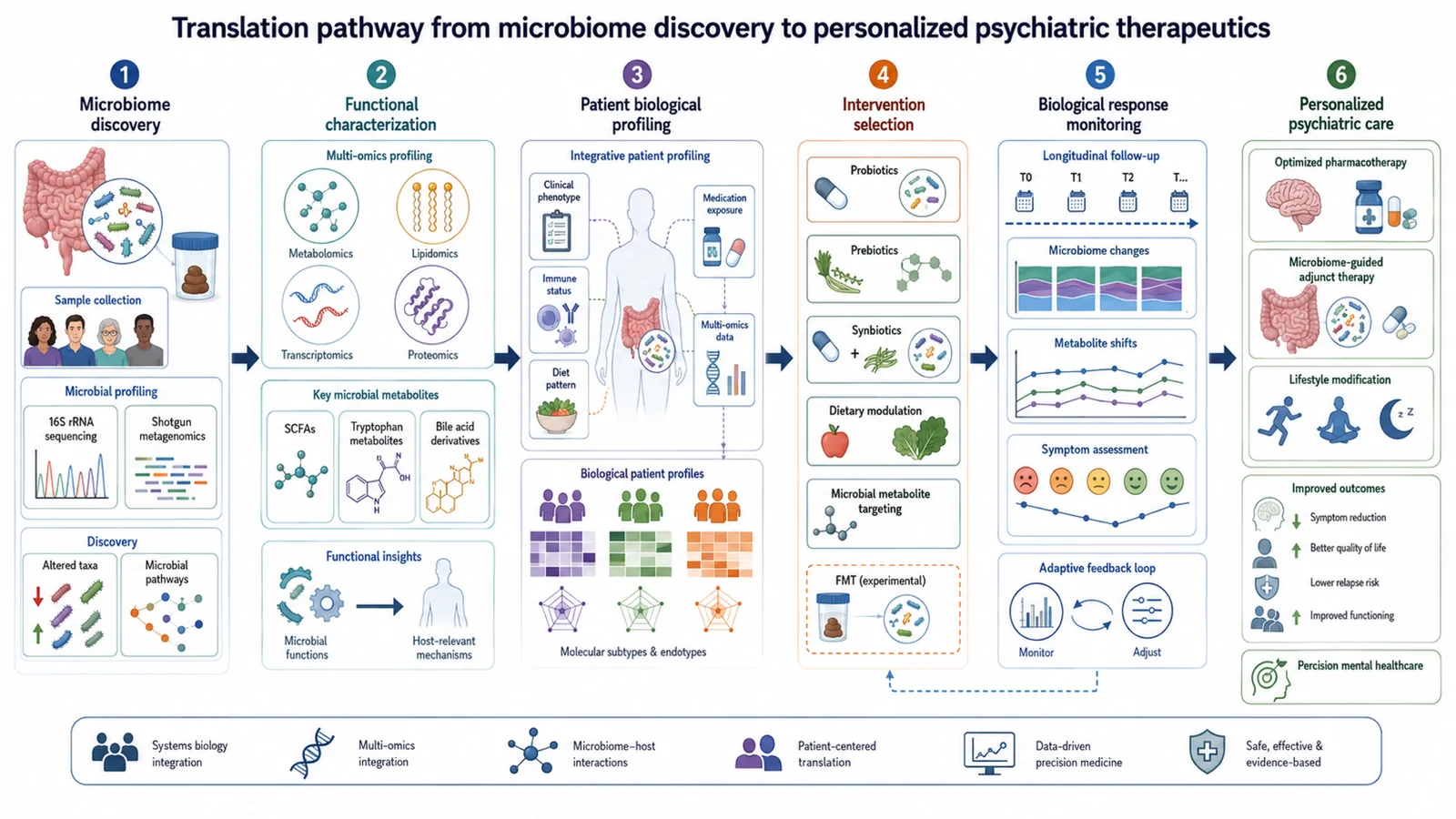
Conceptual future pathway from microbiome discovery to personalized psychiatric therapeutics. This figure illustrates a proposed translational pathway linking microbiome characterization with multi-omics integration, biological stratification, intervention development, and longitudinal monitoring. Microbiome profiling using approaches such as 16S rRNA sequencing and shotgun metagenomics may identify microbial alterations and functional pathways, while integration with metabolomics, lipidomics, transcriptomics, immune profiling, and clinical phenotyping may improve characterization of host-microbiome interactions. These data could inform future evaluation of microbiome-targeted strategies, including probiotics, prebiotics, synbiotics, dietary modulation, microbial metabolite targeting, and experimental approaches such as fecal microbiota transplantation. The pathway is conceptual and does not represent an established treatment algorithm or validated standard of psychiatric care; the proposed stages require further clinical evaluation, standardization, and prospective validation.

Despite substantial progress, several barriers currently limit clinical translation. Interindividual differences in baseline microbiome composition, diet, medication exposure, metabolic status, and psychiatric phenotype may contribute substantially to variability in treatment response, while most intervention studies remain relatively small and provide limited mechanistic validation. Future trials should therefore prioritize adequately powered randomized, placebo-controlled designs where feasible, together with standardized dietary protocols or prospectively monitored dietary intake to minimize diet-related confounding. Longitudinal microbiome sampling at baseline, during intervention, and after treatment should be integrated with clinical outcomes to distinguish transient microbial changes from sustained biological responses. Incorporation of metabolomics and other relevant multi-omics measurements would further help determine whether clinical effects are accompanied by reproducible changes in microbial function and host biological pathways. Prespecified reporting of medication exposure, adherence, adverse events, and baseline microbiome characteristics will also be important for identifying treatment-responsive subgroups and improving reproducibility across studies.

### 3.10 Challenges, knowledge gaps, and future directions for microbiome-driven precision psychiatry in China and global healthcare

Despite rapid advances in microbiome psychiatry, translation of microbiome-based discoveries into clinically applicable precision psychiatry remains challenging. The field has progressed from identifying microbial alterations associated with psychiatric phenotypes toward developing multi-omics frameworks, predictive models, and microbiome-targeted interventions. However, substantial biological, methodological, computational, and clinical barriers must be addressed before microbiome-informed approaches can be integrated into routine psychiatric care. These challenges are particularly relevant in China and other rapidly developing healthcare systems, where large patient populations and expanding biotechnology infrastructure provide unique opportunities for innovation but also require careful validation and standardization.

One of the fundamental challenges in microbiome psychiatry is the reproducibility and generalizability of microbial signatures across populations. Gut microbial composition is shaped by geography, dietary patterns, ethnicity, age, sex, medication and antibiotic exposure, lifestyle, and environmental conditions, making population-specific effects particularly important when interpreting biomarkers developed in Chinese cohorts (6, 7). China itself encompasses substantial regional heterogeneity in staple foods, fiber intake, dietary patterns, and consumption of traditional fermented foods, together with differences in antibiotic exposure, urbanization, environmental conditions, and healthcare practices. Broader real-world clinical evidence also illustrates substantial variation in antimicrobial-treatment exposure and prescribing context across healthcare settings, which may be relevant when considering antibiotic exposure as a potential microbiome confounder (42). These factors may contribute to regional microbiome variation even within the Chinese population and may further limit transferability to populations with different dietary and environmental backgrounds. Consequently, microbial or multi-omics biomarkers developed in Chinese cohorts should not be assumed to generalize directly to European, North American, or other international populations. Independent external validation across geographically and ethnically diverse international cohorts, using harmonized sampling and analytical procedures, will therefore be essential before such biomarkers can be considered broadly applicable for psychiatric diagnosis, stratification, or treatment prediction.

A second major limitation is the predominance of cross-sectional study designs. Most human microbiome psychiatry studies compare individuals with psychiatric disorders and healthy controls at a single time point, making it difficult to determine causal relationships. Whether microbiome alterations contribute to disease development, emerge as a consequence of psychiatric illness, or reflect behavioural and lifestyle changes remains unresolved. Longitudinal studies following individuals from disease onset through treatment response are essential to distinguish causal microbial drivers from secondary disease-associated alterations. Such studies should incorporate repeated microbiome measurements together with clinical trajectories, dietary information, medication exposure, and biological markers.

Methodological heterogeneity represents another important obstacle to clinical translation. Differences in sample collection, storage procedures, DNA extraction methods, sequencing platforms, bioinformatics pipelines, and statistical approaches can produce substantial variability between studies. The lack of standardized protocols limits comparison between datasets and reduces confidence in identified biomarkers. Although initiatives such as standardized microbiome reporting frameworks have improved methodological transparency, further harmonization is required, particularly for clinical trials intended to support regulatory approval or clinical implementation (43). Establishing internationally accepted standards for microbiome sampling, sequencing, data processing, and reporting will be critical for advancing microbiome psychiatry.

Another major knowledge gap concerns the mechanisms linking microbial alterations with psychiatric phenotypes. Although the microbiota-gut-brain axis provides a biologically plausible framework involving immune regulation, microbial metabolites, endocrine signaling, and neural pathways, direct causal evidence in humans remains limited. As broader mechanistic context, studies in human cellular systems demonstrate that lipopolysaccharide-responsive inflammatory signaling can be substantially modulated at the cellular level (44), supporting the biological plausibility of inflammation-related host responses while not constituting direct evidence for psychiatric microbiome mechanisms. Preclinical models, considered solely as mechanistic context rather than as part of the included human evidence, support the biological plausibility that microbiota manipulation can influence stress-related behavior and neurobiological pathways. However, translating findings from experimental models to human psychiatric disorders remains challenging because human mental health is influenced by complex interactions among biological, psychological, social, and environmental factors.

The complexity of psychiatric disorders themselves represents an additional challenge. Current diagnostic systems categorize disorders primarily according to symptoms rather than biological mechanisms. Major depressive disorder, for example, includes individuals with highly variable clinical presentations, treatment responses, inflammatory states, metabolic profiles, and stress histories. A single microbiome signature is therefore unlikely to represent all patients with depression or anxiety. Future research should prioritize biologically informed subtyping approaches that integrate microbiome profiles with metabolomics, immune markers, neuroimaging, genetics, and clinical characteristics. Such multidimensional models may enable identification of patient subgroups with shared biological mechanisms and improved therapeutic responsiveness (20).

The development of microbiome-based predictive models using artificial intelligence introduces both opportunities and challenges. Machine-learning approaches can identify complex patterns within high-dimensional microbiome datasets and may improve prediction of disease status or treatment response. However, many existing models have been developed using relatively small datasets and lack external validation. Overfitting, limited interpretability, and inconsistent feature selection remain significant concerns. For AI-based microbiome psychiatry to become clinically useful, future studies must incorporate large multicenter datasets, transparent modelling approaches, and prospective validation demonstrating improved clinical decision-making compared with existing psychiatric assessment methods.

China represents a particularly important environment for advancing microbiome-driven precision psychiatry. The country has a large and diverse population, extensive clinical research infrastructure, rapidly expanding sequencing capacity, and increasing investment in precision medicine technologies. Clinical cohorts conducted in China have contributed evidence across several areas of microbiome psychiatry, including depression, anxiety, antidepressant response, and multi-omics integration (9, 19, 20). However, future progress will require broader multicenter collaboration across different geographic regions of China to account for dietary diversity, regional environmental differences, and population-specific microbial characteristics.

The integration of microbiome-informed precision psychiatry into routine clinical practice will also require consideration of feasibility, accessibility, and health-system value. High-throughput sequencing and multi-omics profiling remain relatively costly and depend on specialized laboratory and computational infrastructure that may not be consistently available outside major academic or tertiary-care centers. Clinical implementation will therefore require not only technically robust biomarkers but also evidence that microbiome testing provides sufficient incremental clinical benefit to justify its costs compared with existing psychiatric assessment and treatment pathways. Laboratory workflows will need to become standardized, scalable, and sufficiently rapid for routine clinical decision-making, while psychiatrists and other healthcare professionals will require appropriate training to interpret microbiome and multi-omics results and understand their limitations. Reimbursement represents an additional barrier because coverage mechanisms for microbiome-based diagnostics, predictive assays, and personalized interventions remain insufficiently established. Future translation will therefore depend on cost-effectiveness evaluation, accessible laboratory platforms, clinician education, clinically interpretable reporting, appropriate reimbursement pathways, and collaboration among healthcare systems, laboratories, biotechnology developers, regulatory agencies, and payers (34).

Ethical, regulatory, and data governance issues represent additional considerations for future implementation. Microbiome datasets contain complex biological information that may interact with genetic, lifestyle, and health-related data. Ensuring appropriate data protection, responsible artificial intelligence development, and equitable access to microbiome-based technologies will be important as precision psychiatry advances. Furthermore, regulatory frameworks will need to establish standards for microbiome-derived diagnostics, probiotic therapeutics, and personalized microbiome interventions.

Future research priorities should therefore focus on several interconnected directions. First, large-scale longitudinal cohorts integrating microbiome sequencing, metabolomics, immune profiling, genetics, and clinical outcomes are required to establish causal relationships and clinically meaningful biomarkers. Second, standardized sampling and analytical frameworks should be adopted to improve reproducibility across studies and centres. Third, biomarkers and predictive models developed in Chinese or other population-specific cohorts should undergo rigorous external validation in independent international populations before claims of broader clinical generalizability are made. Fourth, machine-learning approaches should be combined with mechanistic biological validation and prospective assessment of clinical utility rather than used solely as predictive tools. Finally, intervention trials should progress toward personalized strategies guided by patient-specific microbiome, metabolic, dietary, and clinical characteristics.

## 4 Discussion

The central implication of this scoping review is that the major translational challenge in microbiome psychiatry is no longer simply the detection of microbiome differences between psychiatric and healthy populations, but determining which signals are reproducible, functionally informative, and clinically actionable. The limited consistency of disorder-specific taxonomic signatures suggests that microbiome associations are strongly dependent on host, environmental, treatment, and methodological context rather than representing universal disease markers (6, 7). This observation strengthens the rationale for shifting attention toward functional microbial pathways and integrated host-microbiome profiles, while also emphasizing that greater biological complexity does not itself guarantee improved clinical utility. Accordingly, microbiome information is currently better viewed as a complementary systems-level source of biological information than as an independent diagnostic framework for depression or anxiety (9, 19).

The increasing application of multi-omics approaches represents a critical advancement in addressing the limitations of early microbiome research. Integration of metagenomics, metabolomics, lipidomics, transcriptomics, and immune profiling provides a more comprehensive understanding of how microbial communities interact with host physiology. Recent multi-omics studies in depression have demonstrated that microbiome alterations are accompanied by metabolic disturbances and changes in biological pathways associated with treatment response, suggesting that microbiome-derived information may capture clinically meaningful biological variation (20, 21). This transition from microbial association to functional interpretation represents a major step toward precision psychiatry because treatment decisions may ultimately be guided by biological characteristics rather than clinical symptoms alone. The findings also highlight the importance of microbial metabolites as potential mediators of psychiatric outcomes. Molecules produced or modified by gut microorganisms, including short-chain fatty acids, tryptophan metabolites, bile acid derivatives, and other bioactive compounds, provide plausible mechanisms through which intestinal microbial ecosystems influence brain function (32). These metabolites may regulate immune activity, neuroendocrine signalling, neurotransmitter pathways, and neuronal plasticity. However, although mechanistic evidence from experimental models is strong, human studies remain insufficient to establish whether specific microbial metabolites are causal drivers of psychiatric disease or biomarkers reflecting underlying biological changes. Psychotropic medication represents an additional challenge when interpreting psychiatric microbiome signatures. Drug-microbiome interactions are bidirectional: antidepressants and other CNS-active medications may alter microbial composition and metabolic activity, while microbial enzymes and metabolites may in turn influence drug metabolism, bioavailability, and therapeutic response. Consequently, microbial differences observed in medicated psychiatric populations cannot automatically be attributed to disease biology alone. Greater use of medication-naïve cohorts, detailed characterization of psychotropic exposure, and longitudinal sampling before and after treatment will be necessary to distinguish disease-associated signatures from medication-induced microbial changes and to clarify potential pharmacomicrobiomic contributions to treatment variability (45).

Indole-3-propionic acid (IPA), a microbiota-derived tryptophan metabolite, has also emerged as a potential neuroprotective and immunomodulatory molecule. However, mechanistic evidence remains predominantly preclinical; for example, IPA supplementation reduced microglial activation and NLRP3 inflammasome signalling in a rat model of traumatic brain injury (46). These findings provide mechanistic context for microbiota-derived indole signaling but should not be interpreted as direct evidence of an antidepressant or anxiolytic mechanism in humans.

An important implication of the reviewed evidence is that microbiome psychiatry may help address one of the major limitations of current psychiatric medicine: biological heterogeneity. Disorders such as MDD and anxiety disorders are clinically defined but biologically diverse. Patients with similar symptoms may differ substantially in inflammatory status, metabolic function, stress-response pathways, and treatment responsiveness. Multi-omics microbiome approaches provide an opportunity to identify biologically meaningful subgroups that may respond differently to therapeutic interventions. This concept aligns with the broader objective of precision psychiatry, where molecular information complements traditional clinical assessment to improve diagnosis, prognosis, and treatment selection (20). The potential role of microbiome-derived biomarkers in treatment prediction represents one of the most clinically relevant areas of development. Current antidepressant treatment selection remains largely based on trial-and-error approaches, resulting in delayed therapeutic response and substantial treatment failure rates. Studies investigating microbiome characteristics associated with antidepressant response suggest that microbial profiles and microbiome-associated metabolic pathways may provide predictive information regarding treatment outcomes (20, 22). However, these findings should be interpreted cautiously because most available studies remain exploratory, with limited sample sizes and insufficient external validation. Before microbiome biomarkers can influence clinical decision-making, they must demonstrate consistent performance across independent populations and provide measurable improvement over existing clinical predictors.

Interpretation and clinical translation of microbiome and multi-omics signatures also require rigorous consideration of confounding and analytical heterogeneity. Diet, psychotropic and antibiotic exposure, lifestyle, body mass index and metabolic status, geography, demographic characteristics, and comorbid conditions can independently influence microbial composition and may therefore generate or obscure apparent disease-associated signatures. Multi-omics integration introduces additional challenges because microbiome, metabolomic, lipidomic, transcriptomic, immune, and clinical datasets differ in dimensionality, analytical platforms, normalization procedures, and biological timescales. Small sample sizes relative to the number of measured features, batch effects, incomplete harmonization across platforms, and limited longitudinal sampling further constrain reproducibility and generalizability. Consequently, clinically useful multi-omics signatures will require standardized pre-analytical and analytical procedures, sufficiently powered multicenter cohorts, transparent data-integration pipelines, and validation across independent populations rather than reliance on discovery-cohort performance alone.

The integration of artificial intelligence (AI) provides important opportunities for identifying complex patterns within high-dimensional microbiome and multi-omics datasets, but current applications remain predominantly exploratory. A major concern is the imbalance between relatively small cohort sizes and very large numbers of microbial and molecular features, which increases susceptibility to overfitting, unstable feature selection, and optimistic estimates of predictive performance. Limited model interpretability further complicates biological inference and clinical acceptance, particularly when predictive features cannot be linked to reproducible biological mechanisms. External validation remains essential because models developed within a single cohort may perform poorly when applied across populations that differ in diet, geography, medication exposure, ethnicity, sequencing platforms, or clinical characteristics. Beyond technical validation, clinical implementation will require prospective demonstration of added value over established psychiatric assessment, transparent and auditable modelling procedures, standardized data processing, and appropriate regulatory and data-governance frameworks. Until these requirements are met, AI-based microbiome models should be regarded primarily as tools for biological discovery and hypothesis generation rather than established clinical decision-support systems.

Microbiome-targeted interventions represent the next step in translating biological discoveries into therapeutic applications. Current evidence from probiotic and psychobiotic trials suggests that microbiome modulation may influence depressive and anxiety symptoms, particularly when used as an adjunctive strategy (23, 24). However, current intervention studies remain heterogeneous regarding microbial strains, treatment duration, patient selection, and outcome measures. Accordingly, precision microbiome intervention should extend beyond generalized probiotic or prebiotic supplementation toward strategies selected according to baseline microbial function, metabolic phenotype, diet, and host characteristics. Emerging approaches include structure-guided or engineered prebiotic polysaccharides designed to preferentially support specific functional microbial populations, microbiota-informed dietary strategies, targeted modulation of microbial metabolites, and multi-omics-guided combination interventions matched to individual biological profiles (40, 47, 48). These approaches provide a conceptual pathway toward mechanism-informed microbiome therapeutics, but most remain at an early translational stage and require rigorous prospective validation before application to individualized psychiatric care. China represents a particularly important contributor to the future development of microbiome precision psychiatry. Chinese research groups have generated substantial evidence in microbiome studies of depression and anxiety, including clinical cohorts investigating microbial signatures, treatment response, and multi-omics interactions (9, 19, 20). The country's large population, diverse dietary patterns, expanding healthcare infrastructure, and strong investment in biotechnology provide unique opportunities for developing large-scale microbiome datasets and precision medicine platforms. However, successful translation will require multicentre collaboration across different regions of China to address population diversity and ensure that microbiome biomarkers are robust and clinically generalizable.

Several limitations of both the current evidence base and the present scoping review should be acknowledged. At the evidence level, most human studies remain observational or cross-sectional, limiting causal interpretation, while substantial heterogeneity in participant characteristics, psychiatric phenotypes, medication and dietary exposure, microbiome sampling, sequencing platforms, bioinformatics pipelines, outcome definitions, and statistical approaches reduces comparability across studies. The available literature may also be affected by publication and selective-reporting bias, because positive or novel microbiome associations may be more likely to be reported than null or inconsistent findings. The present scoping review also has methodological limitations. The literature search was primarily based on PubMed/MEDLINE and PubMed Central, supplemented by Google Scholar and backward citation searching; consequently, the absence of additional bibliographic databases such as Embase, Scopus, Web of Science, and PsycINFO may have resulted in relevant studies being missed. Formal inter-reviewer agreement statistics were not calculated during study selection, and therefore the consistency of screening decisions could not be quantified using measures such as Cohen's *κ*. Therefore, microbiome-based precision psychiatry should be viewed as a complementary biological framework rather than a replacement for comprehensive psychiatric evaluation. Future research should prioritize several key directions. Large-scale longitudinal cohorts integrating microbiome sequencing, metabolomics, immune profiling, genetics, neuroimaging, and clinical outcomes are required to establish causal relationships and clinically meaningful biomarkers. Standardized microbiome analysis protocols should be adopted to improve reproducibility across studies. AI models should undergo rigorous external validation and incorporate mechanistic biological interpretation. Finally, clinical trials should transition toward personalized intervention strategies where treatment selection is guided by patient-specific microbiome and metabolic profiles.

Moving forward, progress in microbiome-driven precision psychiatry will depend on shifting from discovery-oriented associations toward reproducible and clinically actionable frameworks. This transition will require multidisciplinary collaboration among psychiatrists, microbiologists, neuroscientists, nutrition scientists, bioinformaticians, computational researchers, clinical laboratories, and regulatory stakeholders. Standardized procedures for participant characterization, dietary and medication assessment, biospecimen collection, microbiome and multi-omics profiling, data processing, and outcome reporting will be essential for improving reproducibility across studies. Equally important, candidate biomarkers, predictive models, and microbiome-guided interventions must undergo rigorous external and prospective validation across independent and geographically diverse populations before clinical implementation. Ultimately, the value of microbiome-informed precision psychiatry will depend not on the identification of additional microbial associations alone, but on demonstrating that integrated biological information can provide clinically actionable improvements in patient stratification, treatment selection, monitoring, and outcomes beyond conventional psychiatric assessment.

## Conclusion

Microbiome-driven precision psychiatry is shifting from descriptive taxonomic associations toward functional host-microbiome profiling, multi-omics integration, and individualized intervention strategies. However, microbiome-derived biomarkers remain investigational, with limited independent and prospective validation, and should not be considered ready for routine psychiatric implementation until their reproducibility, clinical validity, and added utility beyond conventional assessment have been demonstrated. Future progress will require standardized methodologies, multidisciplinary collaboration, longitudinal multi-omics studies, and rigorous external and prospective validation demonstrating that microbiome-derived information provides clinically actionable improvements in patient stratification, treatment selection, and monitoring beyond conventional psychiatric assessment.

## Data Availability

The data supporting the conclusions of this review are included in the article and its Supplementary Material. This review synthesizes previously published studies and generated no new primary datasets.

## References

[1] Collaborators GMD. Global, regional, and national burden of 12 mental disorders in 204 countries and territories, 1990–2019: a systematic analysis for the global burden of disease study 2019. Lancet Psychiatry. (2022) 9(2):137–50. 10.1016/S2215-0366(21)00395-3 35026139 PMC8776563

[2] Fernandes BS Williams LM Steiner J Leboyer M Carvalho AF Berk M. The new field of ‘precision psychiatry’. BMC Med. (2017) 15(1):80. 10.1186/s12916-017-0849-x 28403846 PMC5390384

[3] Insel TR Cuthbert BN. Brain disorders? Precisely. Science. (2015) 348(6234):499–500. 10.1126/science.aab2358 25931539

[4] Guo H Pan L Wu Q Wang L Huang Z Wang J. Type 2 diabetes and the multifaceted gut-X axes. Nutrients. (2025) 17(16):2708. 10.3390/nu17162708 40871736 PMC12389143

[5] Cryan JF Dinan TG. Mind-altering microorganisms: the impact of the gut microbiota on brain and behaviour. Nat Rev Neurosci. (2012) 13(10):701–12. 10.1038/nrn3346 22968153

[6] Morais LH Schreiber IVHL Mazmanian SK. The gut microbiota–brain axis in behaviour and brain disorders. Nat Rev Microbiol. (2021) 19(4):241–55. 10.1038/s41579-020-00460-0 33093662

[7] Cryan JF O'Riordan KJ Cowan CSM Sandhu KV Bastiaanssen TFS Boehme M. The microbiota-gut-brain axis. Physiol Rev. (2019) 99(4):1877–2013. 10.1152/physrev.00018.2018 31460832

[8] Dalile B Van Oudenhove L Vervliet B Verbeke K. The role of short-chain fatty acids in microbiota–gut–brain communication. Nat Rev Gastroenterol Hepatol. (2019) 16(8):461–78. 10.1038/s41575-019-0157-3 31123355

[9] Jiang H Ling Z Zhang Y Mao H Ma Z Yin Y. Altered fecal microbiota composition in patients with major depressive disorder. Brain Behav Immun. (2015) 48:186–94. 10.1016/j.bbi.2015.03.016 25882912

[10] Zheng P Zeng B Zhou C Liu M Fang Z Xu X. Gut microbiome remodeling induces depressive-like behaviors through a pathway mediated by the host's Metabolism. Mol Psychiatry. (2016) 21(6):786–96. 10.1038/mp.2016.44 27067014

[11] Valles-Colomer M Falony G Darzi Y Tigchelaar EF Wang J Tito RY. The neuroactive potential of the human gut microbiota in quality of life and depression. Nature Microbiology. (2019) 4(4):623–32. 10.1038/s41564-018-0337-x 30718848

[12] Fan Y Pedersen O. Gut microbiota in human metabolic health and disease. Nat Rev Microbiol. (2021) 19(1):55–71. 10.1038/s41579-020-0433-9 32887946

[13] Franzosa EA Sirota-Madi A Avila-Pacheco J Fornelos N Haiser HJ Reinker S. Gut microbiome structure and metabolic activity in inflammatory bowel disease. Nat Microbiol. (2019) 4(2):293–305. 10.1038/s41564-018-0306-4 30531976 PMC6342642

[14] Lloyd-Price J Mahurkar A Rahnavard G Crabtree J Orvis J Hall AB. Strains, functions and dynamics in the expanded human microbiome project. Nature. (2017) 550(7674):61–6. 10.1038/nature23889 28953883 PMC5831082

[15] Beam AL Kohane IS. Big data and machine learning in health care. Jama. (2018) 319(13):1317–8. 10.1001/jama.2017.18391 29532063

[16] Gupta VK Kim M Bakshi U Cunningham KY Davis JM Lazaridis KN. A predictive index for health status using species-level gut microbiome profiling. Nat Commun. (2020) 11(1):4635. 10.1038/s41467-020-18476-8 32934239 PMC7492273

[17] Peters MD Godfrey C McInerney P Munn Z Tricco AC Khalil H. Chapter 11: scoping reviews. JBI Evid Synth. (2020) 169(7):467–73. 10.46658/JBIRM-20-01 33038124

[18] Tricco AC Lillie E Zarin W O'Brien KK Colquhoun H Levac D. PRISMA extension for scoping reviews (PRISMA-ScR): checklist and explanation. Ann Intern Med. (2018) 169(7):467–73. 10.7326/M18-0850 30178033

[19] Jiang H-Y Zhang X Yu Z-H Zhang Z Deng M Zhao J-H. Altered gut microbiota profile in patients with generalized anxiety disorder. J Psychiatr Res. (2018) 104:130–6. 10.1016/j.jpsychires.2018.07.007 30029052

[20] Wang Y Zhou J Ye J Sun Z He Y Zhao Y. Multi-omics reveal microbial determinants impacting the treatment outcome of antidepressants in major depressive disorder. Microbiome. (2023) 11(1):195. 10.1186/s40168-023-01635-6 37641148 PMC10464022

[21] Zu X Xin J Xie H Xu X Shen Y Wang J. Characteristics of gut microbiota and metabolic phenotype in patients with major depressive disorder based on multi-omics analysis. J Affect Disord. (2024) 344:563–76. 10.1016/j.jad.2023.10.104 37863362

[22] Dong Z Shen X Hao Y Li J Xu H Yin L. Gut microbiome: a potential indicator for predicting treatment outcomes in major depressive disorder. Front Neurosci. (2022) 16:813075. 10.3389/fnins.2022.813075 35937875 PMC9354493

[23] Kazemi A Noorbala AA Azam K Eskandari MH Djafarian K. Effect of probiotic and prebiotic vs. placebo on psychological outcomes in patients with major depressive disorder: a randomized clinical trial. Clin Nutr. (2019) 38(2):522–8. 10.1016/j.clnu.2018.04.010 29731182

[24] Schaub A-C Schneider E Vazquez-Castellanos JF Schweinfurth N Kettelhack C Doll JPK. Clinical, gut microbial and neural effects of a probiotic add-on therapy in depressed patients: a randomized controlled trial. Transl Psychiatry. (2022) 12(1):227. 10.1038/s41398-022-01977-z 35654766 PMC9163095

[25] Romijn AR Rucklidge JJ Kuijer RG Frampton C. A double-blind, randomized, placebo-controlled trial of lactobacillus helveticus and bifidobacterium longum for the symptoms of depression. Aust N Z J Psychiatry. (2017) 51(8):810–21. 10.1177/0004867416686694 28068788 PMC5518919

[26] Miller AH Raison CL. The role of inflammation in depression: from evolutionary imperative to modern treatment target. Nat Rev Immunol. (2016) 16(1):22–34. 10.1038/nri.2015.5 26711676 PMC5542678

[27] Mayer EA Nance K Chen S. The gut–brain axis. Annu Rev Med. (2022) 73:439–53. 10.1146/annurev-med-042320-014032 34669431

[28] Sajid S Huang J Kong S Lai C Tan Z Shao Y. A multi-organ atlas links gut microbial metabolites to systemic redox changes in aging mice. Aging Cell. (2026) 25(3):e70433. 10.1111/acel.70433 41797510 PMC12968584

[29] Sajid S Chen X Sun Y Luo J Zhang B Chen L. A translational *in vitro* to *in vivo* study on chronic arsenic exposure induced pulmonary ferroptosis and multi-omics analysis of gut-lung axis correlation. J Hazard Mater. (2025) 495:139049. 10.1016/j.jhazmat.2025.139049 40614423

[30] Hu E Li T Li Z Su H Yan Q Wang L. Metabolomics reveals the effects of hydroxysafflor yellow A on neurogenesis and axon regeneration after experimental traumatic brain injury. Pharm Biol. (2023) 61(1):1054–64. 10.1080/13880209.2023.2229379 37416997 PMC10332220

[31] Ding N Wu H Hua Y Hua R Li B Xie Y. Gut microbiota-derived isovaleric acid alleviates atrial fibrillation by suppressing GSDME-dependent pyroptosis. Cell Metab. (2026) 38(2):370–87.e10. 10.1016/j.cmet.2025.12.017 41638192

[32] O'Mahony SM Clarke G Borre Y Dinan TG Cryan JF. Serotonin, tryptophan metabolism and the brain-gut-microbiome axis. Behav Brain Res. (2015) 277:32–48. 10.1016/j.bbr.2014.07.027 25078296

[33] Fiorucci S Distrutti E. Bile acid-activated receptors, intestinal microbiota, and the treatment of metabolic disorders. Trends Mol Med. (2015) 21(11):702–14. 10.1016/j.molmed.2015.09.001 26481828

[34] Li X-Y Xiang J Wu F-X Li M. NetAUC: a network-based multi-biomarker identification method by AUC optimization. Methods. (2022) 198:56–64. 10.1016/j.ymeth.2021.08.001 34364986

[35] Hu B Wang X Lu S Ying X. A study of bidirectional control of Parkinson's beta oscillations by basal ganglia. Chaos Soliton Fractals. (2025) 195:116267. 10.1016/j.chaos.2025.116267

[36] Zhou Y Cheng Q Chen J Klimov PB Ren Y Zhou D. Genome-wide characterization of blomia tropicalis allergenome: chromosome-level assembly, bioinformatics insights, and clinical validation of novel allergens. Int Immunopharmacol. (2026) 168:115833. 10.1016/j.intimp.2025.115833 41242272

[37] Nikolova VL Cleare AJ Young AH Stone JM. Updated review and meta-analysis of probiotics for the treatment of clinical depression: adjunctive vs. Stand-alone treatment. J Clin Med. (2021) 10(4):647. 10.3390/jcm10040647 33567631 PMC7915600

[38] Su M Tang T Tang W Long Y Wang L Liu M. Astragalus improves intestinal barrier function and immunity by acting on intestinal microbiota to treat T2DM: a research review. Front Immunol. (2023) 14:1243834. 10.3389/fimmu.2023.1243834 37638043 PMC10450032

[39] Yan Q Qin Q Zhang S Chen F Ru Y Zhong Y. Glial cell nutrient sensing: mechanisms of nutrients regulating Alzheimer's Pathogenesis and precision intervention. Crit Rev Food Sci Nutr. (2026) 66(12):2335–51. 10.1080/10408398.2025.2568606 41044993

[40] Luo P He L Liu Z Yang Q Zhou N Qin J. Dietary fucoidan polysaccharide improved growth performance, antimicrobial immune response and gut health of juvenile macrobrachium rosenbergii through microbiota-gut-brain axis. Aquaculture. (2026) 620:743922. 10.1016/j.aquaculture.2026.743922

[41] Kelly JR Borre Y O' Brien C Patterson E El Aidy S Deane J. Transferring the blues: depression-associated gut microbiota induces neurobehavioural changes in the rat. J Psychiatr Res. (2016) 82:109–18. 10.1016/j.jpsychires.2016.07.019 27491067

[42] Zhuang H-H Chen Q-H Wang W Qu Q Xu W-X Hu Q. The efficacy of polymyxin B in treating stroke-associated pneumonia with carbapenem-resistant gram-negative bacteria infections: a multicenter real-world study using propensity score matching. Front Pharmacol. (2025) 16:1413563. 10.3389/fphar.2025.1413563 40183094 PMC11965127

[43] Knight R Vrbanac A Taylor BC Aksenov A Callewaert C Debelius J. Best practices for analysing microbiomes. Nat Rev Microbiol. (2018) 16(7):410–22. 10.1038/s41579-018-0029-9 29795328

[44] Wang J Luo J Rotili D Mai A Steegborn C Xu S. SIRT6 protects against lipopolysaccharide-induced inflammation in human pulmonary lung microvascular endothelial cells. Inflammation. (2024) 47(1):323–32. 10.1007/s10753-023-01911-5 37819455

[45] Xu F Xie Q Kuang W Dong Z. Interactions between antidepressants and intestinal microbiota. Neurotherapeutics. (2023) 20(2):359–71. 10.1007/s13311-023-01362-8 36881351 PMC10121977

[46] Zhao Y Zhou L Xu X Zou J Hu K Quan L. Gut-derived indole-3-propionic acid protects against neuroinflammation after traumatic brain injury through the NLRP3 signaling pathway. Pak Vet J. (2025) 45(2):693–702. 10.29261/pakvetj/2025.173

[47] Medoro A Castagnetti A Intrieri M Scapagnini G Davinelli S. Diet, mycobiome and virome: from mucosal immunity to gut–brain axis regulation. Front Nutr. (2026) 13:1873950. 10.3389/fnut.2026.1873950 42445792 PMC13357866

[48] Yin Y Li Y Zhang X Jia M Zhu S Liu C. Precision prebiotics: engineering food-derived polysaccharides to target specific SCFA-producing taxa for neuroprotection via the microbiota-gut-brain axis. Current Research in Food Science. (2026) 13:101471. 10.1016/j.crfs.2026.101471 42381725 PMC13316636

