# Supplementary material for: Microbiome-driven precision psychiatry: integrating multi-omics, artificial intelligence, and personalized interventions along the gut-brain axis
Source: Front Med (Lausanne). 2026 Oct 15;13:1957715. doi: 10.3389/fmed.2026.1957715 (PMC13716581; doi:10.3389/fmed.2026.1957715)
Supplement: Supplementary file 1 [file Table2.docx]

**Supplementary Table S2.** Detailed characteristics of included human microbiome psychiatry studies

| Study | Country/population | Study design | Psychiatric phenotype | Sample size | Biological sample | Microbiome method | Sequencing platform | Additional omics/analysis | Main contribution |
| --- | --- | --- | --- | --- | --- | --- | --- | --- | --- |
| Jiang et al., 2015 | China; clinically diagnosed MDD and healthy controls | Case-control | MDD | 121 total (58 MDD; 63 HC) | Fecal | 16S rRNA sequencing | Illumina | Diversity/taxonomic analysis | Demonstrated altered gut microbial composition in MDD |
| Jiang et al., 2018 | China; GAD patients and healthy controls | Case-control | GAD | 76 total (40 GAD; 36 HC) | Fecal | 16S rRNA sequencing | NR | Diversity/taxonomic analysis | Demonstrated altered gut microbial profiles associated with GAD |
| Zheng et al., 2016 | China; MDD and healthy controls | Case-control | MDD | 121 total (58 MDD; 63 HC) | Fecal | 16S rRNA sequencing | Illumina | Metabolic/functional analysis | Linked gut microbiome remodeling with depression-related host metabolic changes |
| Valles-Colomer et al., 2019 | European population cohorts | Population-based/clinical cohort analyses | Depression-related outcomes | Large population cohorts; study-specific subsets | Fecal | 16S rRNA and shotgun metagenomics | Illumina/EGA datasets | Functional microbiome analysis | Demonstrated associations between microbial neuroactive potential, quality of life, and depression |
| Dong et al., 2022 | China; Chengdu, Sichuan | Prospective treatment-response cohort | MDD | 93 total (63 MDD; 30 HC) | Fecal + serum | 16S rRNA sequencing | Illumina MiSeq | Metabolomics | Identified microbiome and metabolic features associated with antidepressant response |
| Kazemi et al., 2019 | Iran; Tehran | Double-blind RCT | MDD | 110 randomized; 81 completed | Clinical samples/serum | Microbiome-targeted intervention; microbiome sequencing not central to primary outcome | NR | Tryptophan/kynurenine and BCAA measurements | Evaluated probiotic and prebiotic supplementation for depressive symptoms |
| Schaub et al., 2022 | Switzerland; Basel | Double-blind RCT | Current depressive episode | 60 enrolled; 47 completed | Fecal | 16S rRNA sequencing/quantitative microbiome profiling | NR in main report | Neuroimaging + clinical outcomes | Evaluated probiotic add-on therapy alongside microbiome and neural outcomes |
| Wang et al., 2023 | China; clinical MDD cohort | Treatment-response/multi-omics cohort | MDD | NR in manuscript | Fecal + biological specimens | Microbiome sequencing | NR | Metabolomics/multi-omics | Identified microbial and metabolic determinants associated with antidepressant treatment outcomes |
| Zu et al., 2024 | China; MDD and healthy controls | Case-control/multi-omics study | MDD | 114 total (57 MDD; 57 HC) | Fecal, serum, urine | 16S rDNA sequencing | NR | Serum and urine metabolomics | Identified coordinated microbiome and metabolic phenotypes associated with MDD |
